# Advancing material property prediction: using physics-informed machine learning models for viscosity

**DOI:** 10.1186/s13321-024-00820-5

**Published:** 2024-03-14

**Authors:** Alex K. Chew, Matthew Sender, Zachary Kaplan, Anand Chandrasekaran, Jackson Chief Elk, Andrea R. Browning, H. Shaun Kwak, Mathew D. Halls, Mohammad Atif Faiz Afzal

**Affiliations:** 1 Schrödinger, Inc., New York, 10036 USA; 2 Schrödinger, Inc., Portland, OR 97204 USA; 3 Schrödinger, Inc., San Diego, CA 92121 USA

**Keywords:** Classical molecular dynamics simulations, Organic molecules, Physical properties, Viscosity, Quantitative structure–property relationships, Machine learning

## Abstract

In materials science, accurately computing properties like viscosity, melting point, and glass transition temperatures solely through physics-based models is challenging. Data-driven machine learning (ML) also poses challenges in constructing ML models, especially in the material science domain where data is limited. To address this, we integrate physics-informed descriptors from molecular dynamics (MD) simulations to enhance the accuracy and interpretability of ML models. Our current study focuses on accurately predicting viscosity in liquid systems using MD descriptors. In this work, we curated a comprehensive dataset of over 4000 small organic molecules’ viscosities from scientific literature, publications, and online databases. This dataset enabled us to develop quantitative structure–property relationships (QSPR) consisting of descriptor-based and graph neural network models to predict temperature-dependent viscosities for a wide range of viscosities. The QSPR models reveal that including MD descriptors improves the prediction of experimental viscosities, particularly at the small data set scale of fewer than a thousand data points. Furthermore, feature importance tools reveal that intermolecular interactions captured by MD descriptors are most important for viscosity predictions. Finally, the QSPR models can accurately capture the inverse relationship between viscosity and temperature for six battery-relevant solvents, some of which were not included in the original data set. Our research highlights the effectiveness of incorporating MD descriptors into QSPR models, which leads to improved accuracy for properties that are difficult to predict when using physics-based models alone or when limited data is available.

**Graphical Abstract:**

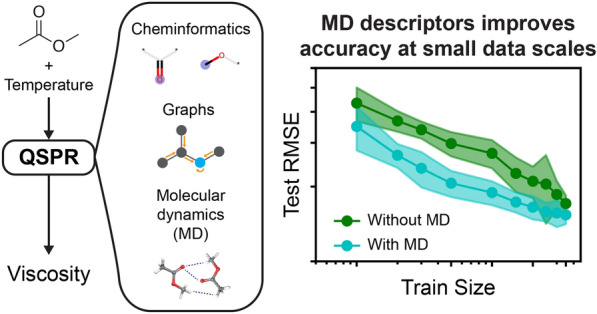

**Supplementary Information:**

The online version contains supplementary material available at 10.1186/s13321-024-00820-5.

## Introduction

Dynamic viscosity, referred to in this work as the viscosity, is an important material property that measures “stickiness”, or a fluid’s resistance to flow when an external force is applied. Viscosity stems from the friction in the bulk caused by adjacent layers of fluid moving at different relative velocities; hence, on the molecular level, viscosity is dictated by intermolecular interactions between particles that lead to internal friction upon fluid flow [1, 2]. Given that viscosity is a fundamental property of fluids, it is often measured in a wide range of applications, such as electronics, pharmaceuticals, and cosmetics [1, 3]. Viscosity is also an important parameter for battery and energy storage research because it dictates the performance of the electrolyte solution within lithium-ion batteries [4, 5]. Thus, accurately and rapidly measuring viscosity is of pivotal importance for the design of new materials.

Experimentally, the viscosity of a fluid can be measured using devices such as rheometers or viscometers [2]. However, measuring a large number of experimental viscosities is challenging, costly, and limited based on the availability of compounds. Alternative to experiments, much effort has been invested in obtaining viscosity using physics-based modeling, such as molecular dynamics (MD) simulations [4, 6, 7]. Despite advancements in simulation procedures, estimating viscosities from MD is especially challenging for highly viscous systems greater than $\sim$5 cP and is computationally expensive, making MD simulations challenging to use for the high-throughput screening of viscosities. Thus, developing computationally efficient and accurate models that can predict the viscosity of molecules is necessary to reduce trial-and-error experimentation or expensive physics-based calculations.

**Fig. 1:**
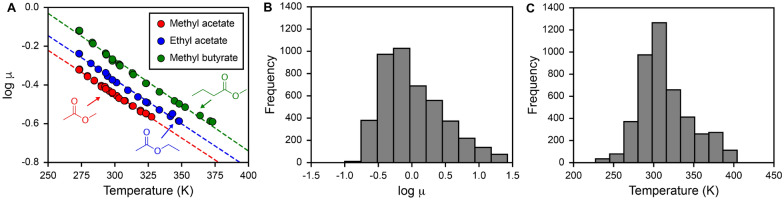
Distribution of the curated viscosity dataset. **A** Log-scale viscosity ($\mu$) in centipoise as a function of temperature of three example battery-relevant structures. Chemical structures are drawn within the plot, and linear dashed lines are included as visual guides. Histogram of **B** log-scale $\mu$ and **C** temperature in Kelvins of the final viscosity dataset consisting of 4440 examples

In contrast to experimental or physics-based methods, data-driven machine learning (ML) approaches can substantially reduce cost and time by learning the underlying connection between molecular structure to bulk properties, such as viscosity, from a large dataset. Fortunately, a substantial amount of experimental viscosity data can be found online or through literature [2, 8], which enables the training of ML models. A variety of ML methods have been used previously to predict viscosity, namely group-contribution-based methods and artificial neural networks (NN) [1–3, 9]. In particular, quantitative structure–property relationships (QSPR)—which correlates molecular-level features to a desired property—have shown great promise in developing accurate viscosity models. For instance, Goussard and others recently developed a ML model that predicts viscosity of pure liquids using a dataset of 300 molecules at a temperature of 25 ^∘^C [2]. While this model is useful for predicting viscosities at room temperature, developing a ML model that can predict the viscosity of molecules across a span of temperatures would broaden its utility. For example, temperature effects on the viscosity of gasoline has a significant impact over fuel efficiency, emphasizing the importance of a viscosity prediction model as a function of temperature [10]. Based on past empirical relationships, such as the Vogel equation [11], the viscosity is expected to be inversely proportional to temperature; hence, increase in temperature results in possibly orders of magnitude decrease in viscosity (see examples in Fig. 1A). An ideal ML model should capture the inverse relationship between viscosity and temperature, which would be useful in various applications, such as consumer packaged goods [2], battery technology [5], or the automobile industry [10].

The recent emergence of deep learning methods have revolutionized how QSPR models are developed. QSPR development was predominantly a traditional cheminformatic task that correlated expert-defined descriptors or fingerprints to a property of interest [12]. The current state-of-the-art deep learning approach is graph neural networks (GNN), specifically graph convolutional networks, which uses convolution operators that learn features directly from a graph representation of a molecule (i.e. representing atoms as nodes and bonds as edges) [13]. GNNs are a promising approach to autonomously create structure–property relationships without having to pre-define descriptors based on expert domain knowledge [14]. However, it is still unclear whether GNNs outperform the descriptor-based models, where the prediction accuracy of both approaches is dependent on the type and size of the data [12]. Furthermore, it is unclear how the inclusion of external features (such as temperature) might impact the prediction accuracy of either descriptor-based descriptors or GNN approaches. Finally, developing accurate QSPR models requires a large, curated viscosity dataset that could broadly generalize viscosity values across a wide range of temperatures. Some recent work has explored the use of data-driven methods to predict viscosities, such as group contribution methods for n-alkanes and iso-alkanes [9] or GNNs for single and binary liquid mixtures [15]. However, the comparison between descriptor-based and graph-based approaches, as well as the inclusion of physics-informed descriptors, has not been well-explored.

In this work, we have extracted and cleaned a large dataset of over 4000 experimental viscosities of small molecules at various temperatures from multiple literature sources. We use this viscosity dataset to build and benchmark machine learning models that can predict viscosity as a function of temperature. We constructed both descriptor-based and GNN-based QSPR models to evaluate whether learned features from graphs could outperform hand-crafted features in predicting viscosities. Additionally, we incorporate information obtained from physics-based simulations into the ML models to further improve the model accuracy. Finally, we employ feature importance analysis tools to evaluate the influence of molecular-based and physics-informed descriptors on QSPR performance. We demonstrate that the developed models are highly accurate and can be used for quick estimation of viscosity of new molecules, which enables these models to be used for the high-throughput screening of viscosities.

## Methods

### Viscosity dataset

We extracted viscosities, temperatures, and structures from the relevant literature and online databases [2, 16–28]. Details of the literature sources are included in Additional file 1: Table S1. All structures were represented as simplified molecular-input line entry-system (SMILES) strings. We curated an initial dataset of 5356 viscosity entries, covering a wide range of temperatures and viscosities. Then, we filtered the dataset using the following steps: (1) filtered for single, organic structures with atomic elements of {H, C, N, O, F, Si, P, S, Cl, Br, and I}; (2) since high experimental errors were observed for high and low extremums of the viscosity and temperature values, the dataset was filtered using the box-and-whisker plot method, where viscosities and temperatures that fall outside of 1.5 times their corresponding interquartile range are removed as outliers; (3) since the viscosity values are expected to be inversely proportional to temperature for bulk liquids, data points that have a positive deviation of viscosity with respect to temperature greater than 0.02 cP were removed as outliers (positive deviations often arise from different literature sources). After applying the data filtration process, we used a total of 4440 viscosity entries for ML model development. This dataset consists of 1005 unique structures, with viscosities ranging from 0.10 cP to 26.52 cP, and temperatures ranging from 227 to 404 K. Since only 136 of the 1005 unique structures have stereoisomers, we did not account for the impact of isomerism in this work. We apply log transform of viscosity to ameliorate the skewed distribution of viscosity values; thus, all viscosities will be presented in the log-scale as log $\mu$, where $\mu$ has units of centipoise.

Figure 1A shows the log-scale viscosity as a function of temperature for three representative small molecules (methyl acetate, ethyl acetate, and methyl butyrate), which are electrolytes relevant to the designing of Li-ion batteries [5]. Figure 1A highlights the inverse proportionality expected between viscosity and temperature, where higher temperature values yield lower viscosities. Figure 1B and C shows the histogram of log-scale viscosity and temperatures for the 4,440 entries respectively. Both Fig. 1B and C shows a right-skewed normal distribution for both log-scale viscosity and temperatures, which means that data is more spread apart at larger viscosity and temperature values. We used the 4,440 viscosity entries to train and evaluate all QSPR models.

### Descriptor-based QSPR models

**Fig. 2:**
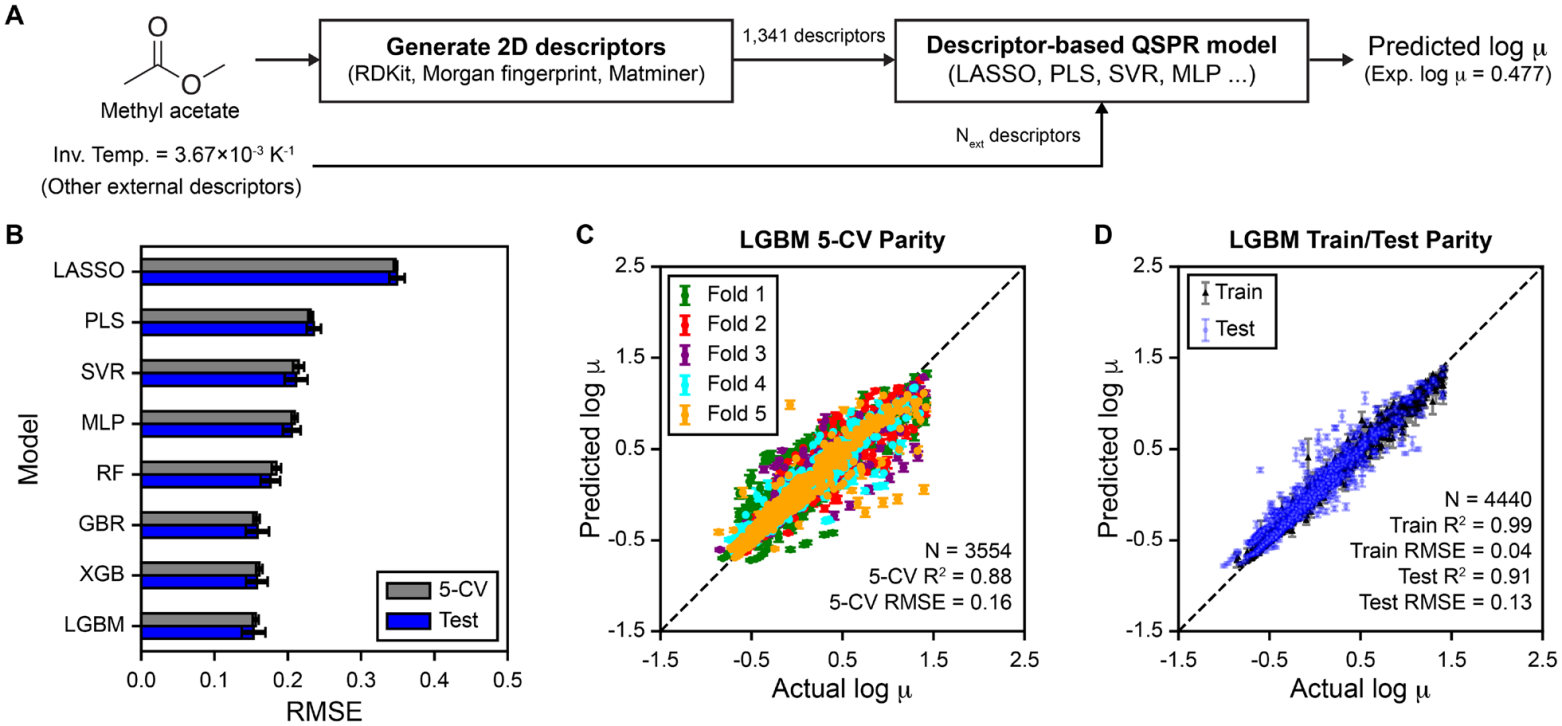
Descriptor-based QSPR approaches for predicting viscosity. **A** Workflow of the descriptor-based approaches using methyl acetate as an example. Methyl acetate is featurized with RDKit, Morgan fingerprint, and Matminer descriptors. A total of 1341 + $N_{ext}$ (external features) features were passed into machine learning model development. The inverse temperature is included in model development to incorporate temperature effects. **B** Five-fold cross validation and test set RMSE for QSPR models. The average RMSE is reported across five out-of-sample train-test splits and the RMSE uncertainty is estimated by computing the standard deviation across the splits. **C** Parity plot between predicted and actual log-viscosity showing the validation set predictions across 5-CV on the training set for a single train/test split when using the LGBM model, which had the highest model score based on Eq. 1. Each color indicates the different validation sets for each of the five folds. The number of examples used (N), $R^2$, and RMSE for 5-CV are reported within the plot. **D** Parity plot between predicted and actual log viscosity for a single 80:20 train:test split for the LGBM model. The total number of examples used (N) and statistics (i.e. $R^2$ and RMSE) for train and test sets are reported within the plot. For all parity plots, a dashed diagonal $y = x$ line is drawn as a guide to indicate which predictions are in agreement with the actual values

The general workflow for developing descriptor-based models is summarized in Fig. 2A. All molecules were featurized with 209 RDKit descriptors, 1000 Morgan fingerprints, and 132 Matminer descriptors. Featurization for RDKit and Morgan fingerprints were implemented using the rdkit package (Version 2021.09.4) [29], whereas Matminer descriptors were implemented using the matminer package (Version 0.6.3) [30]. Based on the Vogel equation of viscosity [11], we expect that log $\mu$ is proportional to the inverse of temperature; hence, we input the inverse of temperature for all ML models. External features, such as experimental inverse temperature or physics-based descriptors, were included as an additional descriptor into the models; hence, a total of 1341 + $N_{ext}$ features were passed into ML model development, where $N_{ext}$ is the number of external features. All features were preprocessed with the following procedure: (1) correlated features with Pearson’s *r* greater than or equal to 0.90 were removed; (2) constant features with variance of zero were removed; and, (3) features were standardized by subtracting the mean and dividing by the standard deviation. On average, 876 of the descriptors remained after feature preprocessing, which were passed as inputs into ML algorithms. Eight different ML algorithms were tested: multilayer perceptron (MLP), support vector regression (SVR), random forest (RF), gradient boosting regression (GBR), light gradient-boosting machine (LGBM) [31], extreme gradient boosting (XGB) [32], least absolute shrinkage and selection operator (LASSO), and partial least squares (PLS). All models were implemented with the scikit-learn package (Version 1.0.2) [33], except LGBM (lightgbm package, Version 3.2.1) and XGB (xgboost package, Version 1.5.1). We selected these ML algorithms based on the current state-of-the-art in the literature to identify the best ML algorithm to predict liquid viscosity [12]. For LASSO models, sparsity or reduction of feature space was applied by modifying the “alpha” parameter in the sklearn module, which dictates the extent of L1 regularization on the coefficients of a linear regression. For SVR models, we used the default radial basis function kernel type in the sklearn module. Hyperparameters for descriptor-based models are described in the Additional file 1: Table S2. For all descriptor-based QSPR models, we used a bagging regressor approach to allow for estimation of prediction errors, where 20 estimators for each ML algorithm were independently trained by randomly sampling the training set with replacement. Prediction values are reported by computing the average prediction of the 20 estimators, and prediction uncertainties are computed using the 90% confidence interval of the prediction values.

### GNN QSPR models

**Fig. 3:**
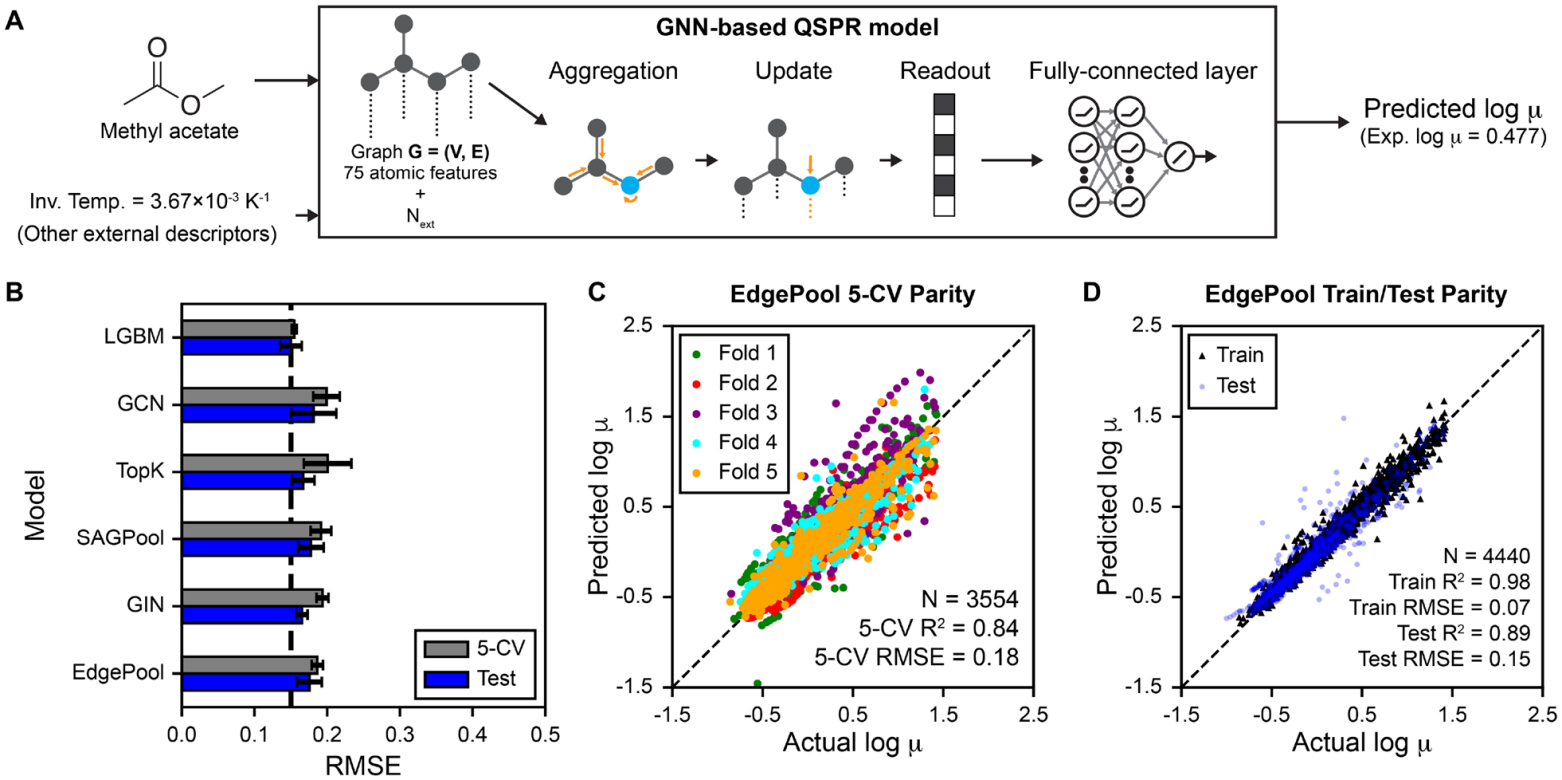
Graph neural network QSPR approaches for predicting viscosity. **A** Workflow of the graph neural network (GNN) based approaches using methyl acetate as an example. Methyl acetate is represented as a molecular graph (G) with atoms as nodes (V) and bonds as edges (E). **B** Five-fold cross validation and test set RMSE for QSPR models. The average RMSE is reported across five random train-test splits and the RMSE uncertainty is estimated by computing the standard deviation across the splits. LGBM is included in this plot as a comparison between the best descriptor-based QSPR model against GNN QSPR models. Only the top five performing GNNs are shown for brevity, which were selected based on Eq. 1. **C** Parity plot between predicted and actual log-viscosity showing the validation set predictions across 5-CV on the training set for a single train/test split when using the EdgePool model, which had the highest model score based on 5-CV and test set $R^2$. Each color indicates the different validation sets for each of the five folds. The number of examples used (N), $R^2$, and RMSE for 5-CV are reported within the plot. **D** Parity plot between predicted and actual log viscosity for a single 80:20 train:test split for the EdgePool model. The total number of examples used (N) and statistics (i.e. $R^2$ and RMSE) for train and test sets are reported within the plot. For all parity plots, a dashed diagonal $y = x$ line is drawn as a guide to indicate which predictions are in agreement with the actual values

GNN models were built using DeepAutoQSAR, Schrödinger’s automated molecular property prediction engine [34, 35]. For GNNs, molecules are treated as molecular graphs with atoms as nodes and bonds as edges, which is illustrated in Fig. 3A. A total of 75 features + $N_{ext}$ (external features) were used to featurize each heavy atom. Atomic featurizations include one-hot encodings of atomic number, implicit valence, formal charge, atomic degree, number of radial electrons, hybridization, and aromaticity [35]. External features were standardized by subtracting the mean and dividing by the standard deviation before being passed into GNNs. For each atom, GNNs aggregate information from its neighboring atoms and update a new atomic vector based on message passing across the molecular graph. The final learned atomic features outputted by the readout phase are then inputted into a fully connected layer to predict log viscosities. Ten graph-based model approaches were evaluated: Graph Convolution Neural Network (GCN) [36], Pytorch version of GCN (TorchGraphConv) [37], TopK [38], GraphSAGE [39], Graph Isomorphism Network (GIN) [40], Self-Attention Graph Pooling (SAGPool) [41], EdgePool [42], GlobalAttention [40], Set2Set [43], and SortPool [44]. Different GNN models differ slightly by how they aggregate information based on successes from previous literature [40, 42]. All graph-based models were trained with PyTorch (Version 1.9.0) [45] for 500 epochs, a learning rate of 0.01, and a dropout ratio of 0.25. Hyperparameters for GNNs are described in the Additional file 1: Table S3.

### Classical molecular dynamics simulations

We performed MD simulations for all the structures at each experimental temperatures in the viscosity dataset to evaluate whether the inclusion of MD descriptors would improve ML models. For all simulations, we used the Schrödinger’s Materials Science Suite (MSS) [46], which leverages the Desmond MD engine to rapidly speed up MD computations through GPU acceleration [7, 47, 48]. All molecules were parameterized with the OPLS4 force field [49]. For each system, we first constructed an amorphous simulation cell with approximately 8000 atoms. The initial density of the system in the amorphous cell structure was 0.5 g/cm^3^.

The equilibration procedure consisted of Brownian minimization of 150 ps, 0.5 ns *NVT* ensemble (Number of atoms, Volume, and Temperature are conserved) with 2 fs time step at temperature of 500 K and pressure of 1 atm, 1 ns *NPT* ensemble (Number of atoms, Pressure, and Temperature are conserved) with 2 fs time step at temperature of 400 K and pressure of 1000 bar, 2 ns *NPT* ensemble with 2 fs time step at temperature of 300 K and pressure of 1 atm, 5 ns *NPT* ensemble with 2 fs time step at the temperature ($T_{exp}$) where experimental viscosity is reported K and pressure of 1 atm, 10 ns *NPT* ensemble with 2 fs time step at $T_{exp}$ and pressure of 1 atm. After this equilibration protocol, we take the average cell size of the last 20% of the previous step and subsequently perform 1 ns *NVT* ensemble with 2 fs time step at $T_{exp}$. The final production run consists of 20 ns *NVT* ensemble with 2 fs time step at $T_{exp}$ with saving a frame at every 100 ps interval.

We extracted eight MD descriptors from the final production MD simulation: packing density (MD_density), percentage free volume (MD_FV), radius of gyration of the molecule (MD_Rg), Hansen solubility parameters (MD_SP, MD_SP_E, and MD_SP_V), heat of vaporization (MD_HV), and root-mean-square displacement (MD_RMSD) (see Additional file 1: Section S2.1 for details). MD descriptors were computed by taking the ensemble-average over the last 10 ns simulation of the production run, and these descriptors show convergence for both low and high viscosity examples (see Additional file 1: Figs. S3 and S4). Averaging MD descriptors using multiple replicas of MD simulations may yield better monotonic trends as a function of temperature, but their values do not significantly differ as compared to descriptors from a single MD simulation (see Additional file 1: Fig. S9). Therefore, we use MD descriptors from a single simulation. These MD descriptors were inputted as external features into the ML models to evaluate whether they could improve the prediction accuracy of viscosities. While MD simulations can yield highly informative descriptors, they also incur additional simulation costs. The estimated computational cost is around one hour per structure and temperature, assuming the use of a computer with a GPU similar to the NVIDIA Tesla T4. However, this cost could be mitigated by employing more efficient GPUs.

### QSPR model training and evaluation

The workflow used to evaluate QSPR models is shown in Additional file 1: Fig. S5. To alleviate the effect of randomness in data splitting, five independent runs with different random seeds were performed with an 80:20 train:test split. Previous literature has used multiple train/test splits to better assess the accuracy of machine learning models [12]. While the average model performance of multiple train/test splits is similar to the model performance when using a single train/test split for predicting viscosity (see Fig. S6 in the Additional file 1), we only report the average model performance of the multiple train/test splits to avoid possible bias in data splitting. Since the viscosity dataset contains multiple entries with the same molecule at different temperature and viscosity values, we implement an out-of-sampling approach for data splitting, where unique compounds are iteratively introduced to the training set until it reaches 80% of the dataset and the remaining 20% of the data is placed in the testing set. Previous studies have observed that out-of-sampling splitting is a better approach to measure model accuracy as compared to random splitting from an application standpoint because model performance from random splitting may lead to over-optimistic model performance for datasets with repeated molecules where the same molecule could appear in both train and test sets [50]. Therefore, all train/test splits in this work uses the out-of-sampling approach such that the test set has unique compounds from the training set.

For each train/test split, a five-fold cross validation procedure (5-CV) was implemented on the training set for hyperparameter tuning and evaluating model generalizability across the training set. In 5-CV, the training set is partitioned into five separate sets, whereby for each of the five folds, one set is left-out as the validation set using the out-of-sample data splitting approach and the remaining sets are used to train the model; this procedure is repeated five times until all of the data instances are within the left-out set exactly once. In this work, we report the 5-CV coefficient of determination ($R^2$) and root-mean-square error (RMSE) of the left-out sets only, which measures the model performance on new compounds. After selecting the best hyperparameters from 5-CV, the model is re-trained with the entire training set and used to predict the test set. The models are evaluated based on their ability to accurately generalize across the training set using the 5-CV approach and predict the testing set, which is summarized by a model score ($\texttt {Score}_M$) in Eq. 1.

1 $$\begin{aligned} \texttt {Score}_M = R^2_{test} \times \left( 1 - \left| R^2_{5-CV} - R^2_{test} \right| \right) \end{aligned}$$

$R^2_{test}$ and $R^2_{5-CV}$ is the coefficient of determination for the test and 5-CV of the train set, respectively. $\texttt {Score}_M$ rewards models that exhibit high generalizability for both the training and testing sets. $\texttt {Score}_M$ penalizes models where the accuracy is low for both sets or when the accuracies between the two sets are very distinct, which may be indicative of overfitting or poor generalization. $\texttt {Score}_M$ is similar to previous model scoring functions in the literature that automatically select good models for structure–property relationships [51]. We primarily use $\texttt {Score}_M$ to rank-order QSPR models based on accuracy on 5-CV and test set prediction accuracy. All QSPR models were implemented using Python (Version 3.8.15).

### Model interpretation

Feature importance was evaluated using the SHapley Additive exPLanations (SHAP) approach (shap package, Version 0.41.0), which is a game theory approach to quantify the contributions of single players in a collaborative game [52, 53]. Shapley values measure the impact of a descriptor to an output property by including or excluding the descriptor across a set of instances. SHAP is a local model-agnostic method for explaining individual predictions. SHAP can also be used as a global interpretation method by aggregations of Shapley values [54]. For all SHAP calculations, we use the test set instances to measure descriptor importance. The average magnitude of Shapley values is reported (i.e. Mean $|$SHAP$|$), and the sign of the importance is determined by computing the Pearson’s *r* correlation coefficient between the Shapley and descriptor values. Positive Pearson’s *r* between Shapley and descriptor values indicate that the feature positively contributes to the output property, whereas negative Pearson’s *r* indicates the converse. Additional details about the SHAP method could be found in previous literature [12, 55, 56].

## Results and discussion

### Performance of descriptor-based QSPR models

We first sought to develop QSPR models using the descriptor-based approach, where hand-crafted two-dimensional (2D) descriptors and fingerprints are used as inputs into the machine learning model. Figure 2A shows the general workflow for inputting hand-crafted descriptors and external descriptors, such as inverse temperature, into QSPR models to predict log viscosities (see Methods for more details). Figure 2B shows the 5-CV and test set RMSE for the eight ML algorithms when using five random, out-of-sample 80:20 train:test splits across the viscosity dataset. ML algorithms were rank-ordered based on their model scores as described in Eq. 1. From Fig. 2B, we observe that tree-based ML models, such as LGBM, XGB, and GBR, were the top performers in predicting log viscosities, followed by other non-linear approaches such as SVR and MLP. Linear models like LASSO and PLS perform the worst, suggesting that a non-linear relationship between the 2D descriptors and log viscosities may be necessary for an accurate model. For all models, 5-CV and test set RMSEs are very similar, which shows that the models’ ability to generalize across the training set is correlative to its ability to generalize to unseen examples.

Since LGBM had the highest model score, we further investigated its accuracy in the 5-CV of the training set and in test set predictions. Figure 2C shows the parity plot between predicted versus actual log viscosities when performing 5-CV across the training set when using the LGBM algorithm; only predictions on the left-out validation set are shown for each of the five cross validation folds. The 5-CV parity plot shows that the majority of the points lie along the diagonal $y = x$ line, suggesting that the LGBM model generalizes well across the training set with a 5-CV $R^2$ of 0.88 and RMSE of 0.16. Figure 2D shows a parity plot of predicted versus actual log viscosities for the training set and testing set when performing an 80:20 train:test split and using the LGBM algorithm. The LGBM model learned the training set well with a train $R^2$ of 0.99 and RMSE of 0.04 and predicted the left-out test set with lower accuracy (i.e. test $R^2$ of 0.91 and RMSE of 0.13). The parity plots in Fig. 2C and D show minimal outliers in the LGBM model predictions, which suggests the model is accurately capturing trends between structure, temperature, and viscosities.

### Performance of GNN QSPR models

We next evaluated whether GNNs might outperform the descriptor-based approaches in predicting temperature-dependent viscosities. Figure 3A shows the general workflow of using GNNs to predict viscosities using methyl acetate as an example (see Methods section for details). Figure 3B shows the 5-CV and test set $R^2$ for the top five GNN models ranked based on model score and the top descriptor-based LGBM model as a comparison. While the EdgePool model had the highest model score, the overall 5-CV and test set $R^2$ is comparable between the different GNN approaches, which suggests that varying GNN architectures did not yield higher accuracy in viscosity predictions. The GNN models have slightly lower 5-CV and test set $R^2$ as compared to the descriptor-based LGBM model (performance is drawn as a vertical dashed line), which suggests that descriptor-based approaches may slightly outperform graph-based approaches for this viscosity dataset. Figure 3C shows the parity plot between predicted versus actual log viscosities when performing 5-CV across the training set when using the EdgePool model. EdgePool achieves a 5-CV $R^2$ of 0.84 and RMSE of 0.18, which is slightly poorer compared to LGBM (see Fig. 2C). Figure 3D shows a parity plot between predicted versus actual log viscosities for an 80:20 train:test split using the EdgePool algorithm. In comparison to LGBM (Fig. 2D), EdgePool achieves a slightly poorer test set $R^2$ of 0.89 and RMSE of 0.15. Overall, these results show that GNNs could be used to predict viscosities; however, descriptor-based approaches perform slightly better for this dataset.

### Impact of molecular simulation derived descriptors on QSPR models for viscosity

**Fig. 4:**
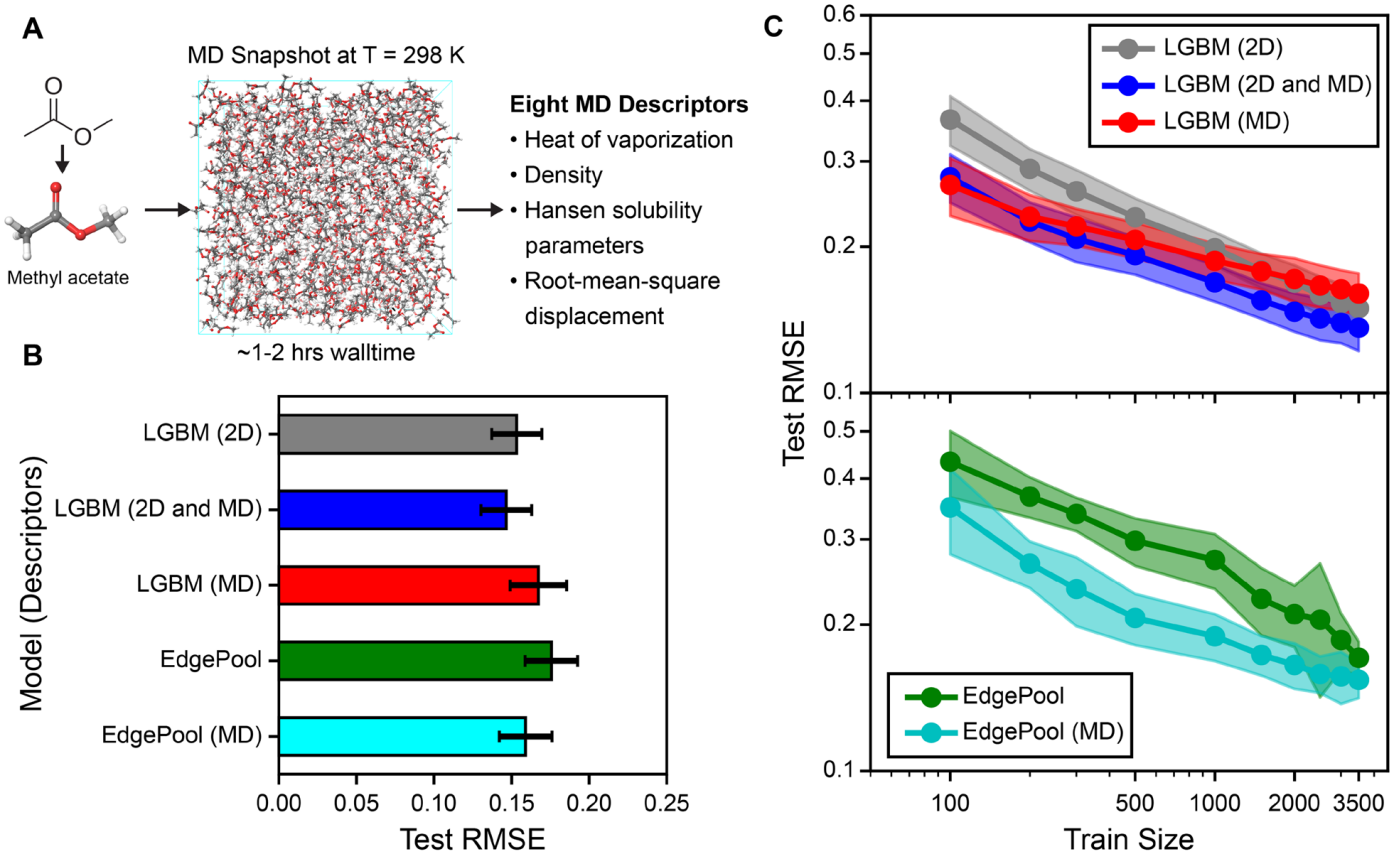
Impact of MD descriptors in QSPR models for viscosity predictions. **A** Simulation snapshot of methyl acetate at T = 298 K, which was used to compute eight MD descriptors. **B** Test set root-mean-square error (RMSE) for descriptor-based LGBM model and GNN-based EdgePool model when including two-dimensional descriptors (2D), molecular dynamics (MD) descriptors, or combinations of 2D and MD (2D and MD) into the QSPR models. The average RMSE is reported across five random, out-of-sample train-test splits and the RMSE uncertainty is estimated by computing the standard deviation across the splits. **C** Log-scale learning curve showing test set RMSE versus train set size when using 20% of the dataset as test set and re-training the models with increasing training set sizes. These curves are plotted for LGBM and EdgePool models with and without MD descriptors. Twenty train-test splits were implemented to obtain accurate measurements of test RMSE, where the mean test set RMSE is reported and the uncertainty is estimated by the standard deviation of the test set RMSEs

We next investigated whether the inclusion of physics-based descriptors computed from molecular dynamics simulations could help improve the QSPR accuracy of temperature-dependent viscosities. We hypothesized that since viscosity is dictated by intermolecular interactions during fluid flow, MD-derived features that capture these interactions may improve QSPR models for viscosity. As an example, Fig. 4A shows a production simulation snapshot of methyl acetate at T = 298 K, which was used to generate eight MD descriptors for QSPR model development (see the Methods section for details). We evaluated the inclusion of MD descriptors for both descriptor-based and GNN-based QSPR models, specifically the LGBM algorithm for the descriptor-based model and the EdgePool algorithm for the graph-based model since these models obtained the highest model score (see Fig. 3B). For LGBM models, inclusion of 2D and MD descriptors would result in 1350 descriptors that consist of 1341 2D descriptors, one inverse temperature descriptor, and eight MD descriptors. For EdgePool models, inclusion of MD descriptors would result in a total of nine external features consisting of one inverse temperature descriptor and eight MD descriptors. All descriptors are preprocessed by correlated and constant feature removal as described in the Methods section. Fig. 4B compares the test set RMSE of LGBM and EdgePool when using either 2D descriptors alone, 2D and MD descriptors together, and MD descriptors alone to predict the log viscosities. We observe that LGBM with 2D and MD descriptors has a slightly lower test set RMSE as compared to LGBM trained with 2D and MD descriptors separately. Similarly, inclusion of MD descriptors for EdgePool slightly decreases test set RMSE as compared to EdgePool alone.

While MD descriptors did not significantly improve test set RMSEs when performing an 80:20 train:test split across the viscosity dataset, we hypothesized that MD descriptors may be more useful in the low-data region where highly informative descriptors are expected to improve prediction accuracy for viscosity. Figure 4C shows the learning curve for LGBM and EdgePool models with the inclusion of 2D descriptors alone, 2D and MD descriptors, and MD descriptors alone. The learning curve measures the effectiveness of these models and varying featurization schemes to predict an unseen test set consisting of 20% of the viscosity dataset when increasing the number of training examples inputted to the models. For LGBM models, we observe that using the combination of 2D and MD descriptors or MD descriptors alone outperform using 2D descriptors alone at the small training sizes ($\sim$100 data points) in predicting the test set RMSE. We observe a similar pattern when training EdgePool with and without MD descriptors, where inclusion of MD descriptors lowers test set RMSE between $\sim$100 to $\sim$1000 training sizes. As the training size increases above $\sim$1000 examples, the prediction accuracy gained from including MD descriptors becomes statistically insignificant when comparing against models without MD descriptors. We further quantified the percent change in test RMSE with and without MD descriptors in the Additional file 1: Fig. S8. We found that both LGBM and EdgePool models achieve at least 15% reduction in test RMSE at 500 training examples and plateauing at 10% reduction in test RMSE at 3,500 examples. These results suggest that MD descriptors are particularly advantageous for viscosity predictions at the low-data regions, but the usefulness of MD descriptors are diminished at the high-data regions since the ML models may better correlate non-linear trends between lower dimensional features (e.g. 2D descriptors) and viscosities.

Figure 4C highlights the surprisingly good performance of LGBM models when using only eight MD descriptors, which outperformed the same model when using more than hundreds of 2D descriptors at the small data scale. The improved model performance suggests that MD descriptors are informative to viscosity predictions, which is further supported by feature importance analysis in the next section. Furthermore, while MD simulations struggle to directly measure high viscosities that are greater than five cP, MD excels in accurately predicting certain properties, such as system density, heat of vaporization, and solubility parameters, which shows a high degree of correlation with experimental data [48, 49, 57]. Thus, we can reliably use these MD descriptors in our ML models even for molecules with high viscosities.

### Model interpretability for descriptors-based QSPR models

**Fig. 5:**
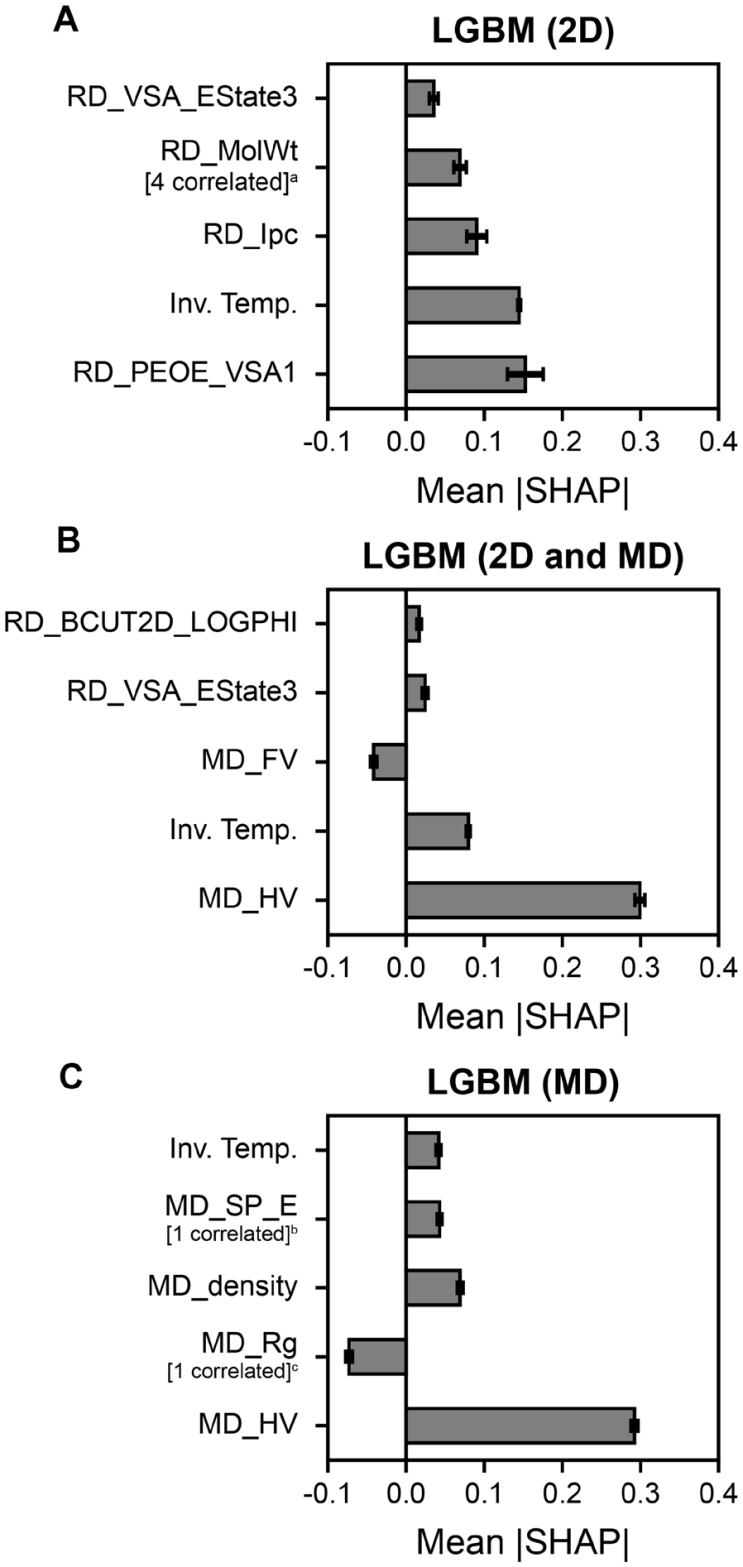
Feature importance of descriptor-based LGBM models. Top 5 important features measured as the average magnitude of SHapley Additive exPLanations (SHAP) values (i.e. Mean |SHAP|) for LGBM models trained with **A** 2D descriptors only, **B** 2D and MD descriptors, and **C** MD descriptors only. Positive Mean |SHAP| indicates that the descriptor positively contributes to viscosity, whereas negative Mean |SHAP| indicates the converse. Descriptors with prefixes of “RD” and “MD” refer to RDKit and MD descriptors, respectively. The average Mean |SHAP| of twenty LGBM estimators is reported and the uncertainty is estimated by the computing standard deviation of the Mean |SHAP| values. The number of features correlated to the top features based on a Pearson’s *r* correlation coefficient cutoff greater or equal to 0.90 are shown in brackets and summarized here (parenthesis is Pearson’s *r* correlation to the top feature): ^a^RD_HeavyAtomMolWt (0.99), RD_ExactMolWt (1.00), RD_Chi0v (0.93), RD_LabuteASA (0.93); ^b^MD_SP (0.93); ^c^MD_RMSD (0.95)

One advantage of descriptor-based QSPR models is the ability to interpret which features are most relevant to predicting viscosity, which remains an active area of research for graph-based QSPR models that are generally more challenging to interpret [58–61]. Given that LGBM models with permutations of 2D and MD descriptors performed similarly in predicting viscosities (see Fig. 4B), we sought to analyze the underlying connections between the descriptors and viscosities to see if there are any similarities when varying the featurization spaces. We use the SHAP approach to quantify feature importance by measuring the impact of each descriptor to viscosity predictions by including or excluding the descriptor across a set of instances (see Methods for details). The SHAP method is advantageous because it is a model-agnostic approach that is capable of quantifying feature importance even for “black box” models, such as deep neural networks [62]. Figure 5 shows the top five features measured by the average magnitude of Shapley values when using the LGBM model with 2D descriptors only, 2D and MD descriptors, and MD descriptors only.

When using 2D descriptors only (see Fig. 5A), “MOE-like” charge van der Waal’s surface area descriptors (RD_PEOE_VSA1), inverse temperature (Inv. Temp.), graph-like descriptors (Ipc) [63], molecular weight (RD_MolWt), and EState VSA Descriptor 3 (RD_VSA_Estate3) are the top descriptors that contribute to predictions of viscosity. We expect experimental temperature to be an important parameter for temperature-dependent viscosity predictions, hence it is no surprise that Inv. Temp. is one of the top descriptors. The other descriptors suggest that molecular size and charge distribution contributes to viscosity, which is consistent with our understanding that larger molecules result in more intermolecular attractions that lead to higher viscosities and charges influence attractiveness between molecules.

When combining 2D and MD descriptors (see Fig. 5B), the top descriptors when using 2D descriptors alone are replaced with two MD descriptors: heat of vaporization (MD_HV) and free volume (MD_FV). MD_HV is computed from nonbonded interactions and is the top descriptor that contributes to viscosity, which agrees with our hypothesis that intermolecular interactions captured from MD simulations may be more informative for a QSPR model as compared to 2D descriptors. Interestingly, experimental heat of vaporization has been previously used as a parameter to correlate with viscosity [64], which is in agreement with the top MD_HV descriptor identified by the LGBM model. MD_FV captures the voids between molecules in solution, which has been observed in the literature to be related to viscosity [9, 65]. MD_FV is also negatively correlated to viscosity (see Fig. S2 in the Additional file 1), which means that smaller voids in solution results in favorable interactions between molecules and, hence, higher internal friction and viscosity. The results in Fig. 5B highlights that MD descriptors are important for viscosity despite being in the presence of more than hundreds of 2D descriptors.

When using MD descriptors only (Fig. 5C), MD_HV remains to be the top descriptor relevant to viscosity predictions consistent when using both 2D and MD descriptors. Information about molecular size, such as radius of gyration (MD_Rg) and density (MD_density), are the next top descriptors when using MD descriptors, which is similar with the top descriptors observed when using 2D descriptors only. Interestingly, experimental inverse temperature is the least important of the top five features when using MD descriptors only, which may be because MD descriptors capture temperature effects during the simulation or use temperature as part of the calculations. Altogether, Fig. 5 suggests that descriptors from MD simulations that capture nonbonded interactions, such as the heat of vaporization, are useful to accurately predict viscosities.

### Temperature-dependent viscosity predictions for battery-relevant solvents

We next evaluated whether the QSPR models can capture the temperature dependence of log viscosities. We focused on six pure solvents previously studied by Logan and coworkers, which were used to potentially improve lithium ion battery electrolytes: methyl acetate (MA), ethyl acetate (EA), methyl butyrate (MB), methyl propionate (MP), dimethyl carbonate (DMC), and ethyl methyl carbonate (EMC) [5]. These pure solvents could be added as co-solvents for lithium ion batteries to lower viscosities and increase electric conductivity; hence, these solvents can improve how fast a battery can charge or discharge. The authors report experimental temperature-dependent viscosities for these six solvents, which were not used in the original data curation of viscosities in this work. However, some of these solvents have been observed in other databases (e.g. PubChem [20]), so there is some overlap between the viscosities from Ref. [5] and the viscosity dataset used in this work. We investigate whether the QSPR models in this work could predict the experimental viscosity trends measured from Ref. [5].

**Fig. 6:**
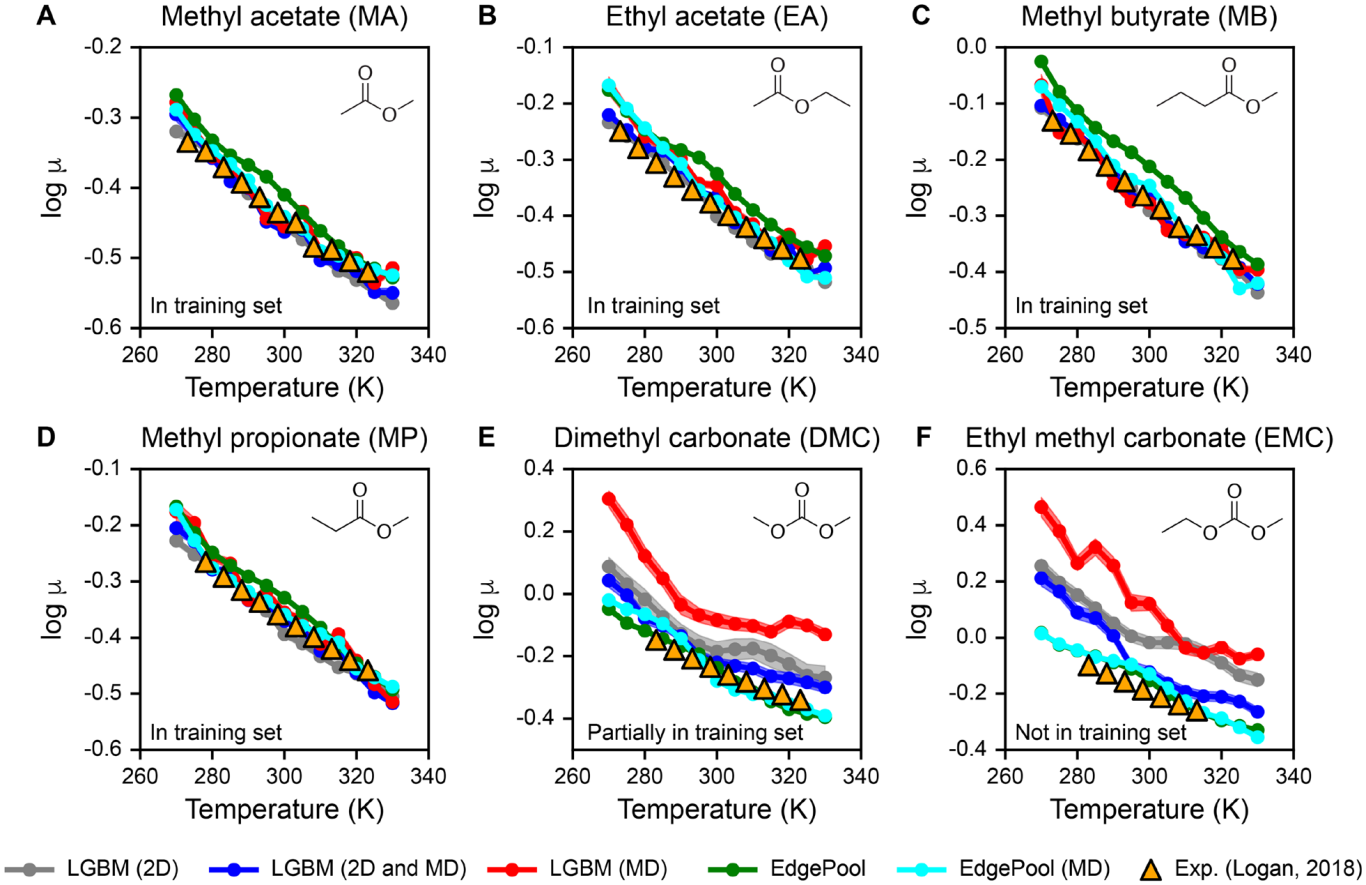
QSPR performance on six battery-relevant solvents. Predictions of descriptor-based LGBM model and GNN-based EdgePool model when using two-dimensional descriptors (2D), molecular dynamics (MD) descriptors, or combinations of 2D and MD (2D and MD) in the QSPR models for six battery electrolytes: **A** methyl acetate (MA); **B** ethyl acetate (EA); **C** methyl butyrate (MB); **D** methyl propionate (MP); **E** dimethyl carbonate (DMC); and **F** ethyl methyl carbonate (EMC). Orange triangles represent experimental viscosities extracted from Ref [5]. MA, EA, MB, and MP are in the training set and contain the temperature ranges that encompass those found in Ref [5]. DMC is partially in the training set such that only two temperatures are provided to the models at T = 293.15 and 298.15 K. EMC is not within the training set at all. Molecular structures are drawn in the upper right of each plot

To eliminate the effect of data splitting, we re-trained the QSPR models using the entire viscosity dataset in this work. Figure 6 shows the log viscosities versus temperature predictions for the six solvents using descriptor-based LGBM and GNN-based EdgePool models with varying featurization inputs (2D descriptors only, 2D and MD descriptors, and MD descriptors only). MA, EA, MB, and MP are structures within the viscosity dataset (i.e. training set) and encompass the same range of temperatures as experimentally measured in Ref. [5]. Hence, across all QSPR models and featurization schemes, the experimental points shown as orange triangles are well-captured for MA, EA, MB, and MP (see Fig. 6A–D). These results show that the QSPR models capture experimental trends from Ref. [5] for structures and temperatures already seen in the training set, suggesting consistency between the viscosity values from Ref. [5] and the viscosity dataset in this work.

For DMC (see Fig. 6E), the solvent is partially within the training set such that only two temperatures at T = 293.15 K and 298.15 K have been seen by the model. Hence, the QSPR models would be extrapolating across a wider range of temperatures between 280 K to 323 K that were experimentally varied in Ref. [5]. We observe that EdgePool with (cyan line) and without MD descriptors (green line), as well as LGBM with 2D and MD descriptors (blue line), can accurately capture the experimental viscosities. Interestingly, predictions from LGBM models with 2D descriptors or MD descriptors alone have the largest deviation from the experimental viscosities, which suggests that combining 2D and MD descriptors helped improve generalizations across temperature. For EMC (see Fig. 6F), the solvent is not within the training set; hence, QSPR models would be predicting on a new molecule. We observe similar trends as in Fig. 6E, where EdgePool with and without MD descriptors accurately capture experimental viscosity trends. LGBM with 2D and MD descriptors outperform models trained with 2D or MD descriptors alone in capturing experimental trends.

Altogether, the predictions on the six battery-relevant solvents show that these QSPR models can: (1) capture the inverse relationship between log viscosity and temperature, (2) predict temperature-dependent viscosities of new structures, and (3) improve in generalizability by inclusion of MD descriptors for descriptor-based LGBM models. Given that the EdgePool model without the inclusion of MD descriptors performed well on battery solvents shown in Figure 6, we use this model to predict log viscosities for other solvents related to battery electrolyte design for lithium metal anodes from Ref. [66]. Viscosity predictions for 50 solvents at temperature ranges between 270 and 330 K are available in the Additional file 1: Section S4.3. Future work will focus on using these models to screen new compounds to identify materials with promising viscosities.

## Conclusion

In this work, we developed quantitative structure–property relationships (QSPR) to predict temperature-dependent viscosities of small organic molecules using a curated dataset of over 4000 experimental viscosities. Both descriptor-based and graph-based models were benchmarked to identify the best machine learning algorithms that could accurately predict experimental viscosities, which were the light gradient-boosting machine (LGBM) algorithm and EdgePool algorithms for descriptor-based and graph-based approaches, respectively. Including molecular dynamics (MD) descriptors slightly improved QSPR models compared to using two-dimensional descriptors alone, suggesting that using features that capture intermolecular interactions can help improve predictions of viscosities. The improvement in prediction accuracy upon inclusion of MD descriptors is most pronounced when training viscosity models using small datasets of less than 1000 examples. Analyzing the top features related to viscosity for the LGBM model reveal that MD descriptors become most important to predicting viscosity, specifically the heat of vaporization that captures nonbonded interactions between molecules. Finally, the QSPR models can accurately capture the inverse relationship between temperature and viscosity for six battery-relevant solvents.

These results demonstrate that regardless of descriptor-based or graph-based models, the inclusion of MD descriptors that capture intermolecular interactions is useful for prediction of viscosities, especially at small data sizes. The usefulness of MD descriptors may be even more relevant for mixture systems, where MD descriptors could more broadly generalize since they are not single-molecule-dependent as compared to two-dimensional structural descriptors. However, one of the drawbacks of using MD descriptors is the computational cost to generate them. The improvement in accuracy from using MD at the small data scale, generalizability of MD descriptors to heterogeneous systems, and generating automated computational workflows may help outweigh the cost of computing these descriptors. Future work will investigate the utility of MD descriptors in predicting viscosities for mixture systems, such as binary mixtures explored in a recent work [15] (Additional file 3).

## Scientific contribution

- Curated a viscosity dataset of more than 4000 examples consisting of small organic molecules and trained quantitative structure property relationships (QSPR) models to accurately predict viscosity as a function of temperature.
- Encoding molecular dynamics (MD) simulation-derived descriptors that capture intermolecular interactions improve viscosity prediction, especially in small data scenarios.
- Feature importance analysis reveal that MD-derived heat of vaporization is found to be the most useful descriptor relevant to viscosity even in the presence of hundreds of two-dimensional descriptors.

### Supplementary Information

**Additional file 1.** This file contains details of the curated viscosity dataset, how molecular dynamics descriptors are computed, the correlation between top descriptors and viscosity, the stability of molecular dynamics descriptors, and hyperparameters for QSPR models.

**Additional file 2.** Viscosity dataset used for generating ML models.

**Additional file 3.** Viscosity predictions for 50 battery-relevant solvents at temperature ranges between 270 K - 330 K.

## Data Availability

Only 3582 of 4440 examples of the viscosity dataset are made available due to copyright restrictions as described in the Additional file 2. The subset viscosity dataset and a pre-trained LGBM model using the subset dataset are provided under the Creative Commons Non-Commercial 4.0 International (CC-BY-NC 4.0) Attribution License. This license allows for the use of the data and the creation of adaptations, exclusively for non-commercial purposes, provided that appropriate credit is given. The additional file contains details of the curated viscosity dataset, how molecular dynamics descriptors are computed, correlation between top descriptors and viscosity, stability of molecular dynamics descriptors, hyperparameters for QSPR models, and availability of the viscosity dataset and model.

## References

[1] Conte E, Martinho A, Matos HA, Gani R (2008). Combined group-contribution and atom connectivity index-based methods for estimation of surface tension and viscosity. Ind Eng Chem Res.

[2] Goussard V, Duprat F, Ploix J-L, Dreyfus G, Nardello-Rataj V, Aubry J-M (2020). A new machine-learning tool for fast estimation of liquid viscosity: application to cosmetic oils. J Chem Inf Model.

[3] Chen Y, Peng B, Kontogeorgis GM, Liang X (2022). Machine learning for the prediction of viscosity of ionic liquid-water mixtures. J Mol Liq.

[4] Dajnowicz S, Agarwal G, Stevenson JM, Jacobson LD, Ramezanghorbani F, Leswing K, Friesner RA, Halls MD, Abel R (2022). High-dimensional neural network potential for liquid electrolyte simulations. J Phys Chem B.

[5] Logan ER, Tonita EM, Gering KL, Li J, Ma X, Beaulieu LY, Dahn JR (2018). A study of the physical properties of li-ion battery electrolytes containing esters. J Electrochem Soc.

[6] Santak P, Conduit G (2020). Enhancing NEMD with automatic shear rate sampling to model viscosity and correction of systematic errors in modeling density: application to linear and light branched alkanes. J Chem Phys.

[7] Mohanty S, Stevenson J, Browning AR, Jacobson L, Leswing K, Halls MD, Afzal MAF (2023). Development of scalable and generalizable machine learned force field for polymers. Sci Rep.

[8] Reid RC, Prausnitz JM, Poling BE (1987). The properties of gases and liquids.

[9] Jovanović JD, Grozdanić ND, Radović IR, Kijevčanin ML (2023). A new group contribution model for prediction liquid hydrocarbon viscosity based on free-volume theory. J Mol Liq.

[10] Zhu Ling, Chen Jiaqing, Liu Yan, Geng Rongmei, Junjie Yu (2012). Experimental analysis of the evaporation process for gasoline. J Loss Prev Process Ind.

[11] Poling BE, Prausnitz JM, O’Connell JP (2000). The properties of gases and liquids.

[12] Jiang D, Zhenxing W, Hsieh C-Y, Chen G, Liao B, Wang Z, Shen C, Cao D, Jian W, Hou T (2021). Could graph neural networks learn better molecular representation for drug discovery? A comparison study of descriptor-based and graph-based models. J Chem.

[13] Reiser Patrick, Neubert Marlen, Eberhard André, Torresi Luca, Zhou Chen, Shao Chen, Metni Houssam, van Hoesel Clint, Schopmans Henrik, Sommer Timo (2022). Graph neural networks for materials science and chemistry. Commun Mater.

[14] Zhenqin W, Ramsundar B, Feinberg EN, Gomes J, Geniesse C, Pappu AS, Leswing K, Pande V (2018). MoleculeNet: a benchmark for molecular machine learning. Chem Sci.

[15] Bilodeau C, Kazakov A, Mukhopadhyay S, Emerson J, Kalantar T, Muzny C, Jensen K (2023). Machine learning for predicting the viscosity of binary liquid mixtures. Chem Eng J.

[16] Saldana DA, Starck L, Mougin P, Rousseau B, Ferrando N, Creton B (2012). Prediction of density and viscosity of biofuel compounds using machine learning methods. Energy Fuels.

[17] Viswanath DS, Ghosh TK, Prasad DHL, Dutt NVK, Rani KY, Viswanath DS, Ghosh TK, Prasad DHL, Dutt NVK, Rani KY (2007) Correlations and estimation of pure liquid viscosity. In: Viscosity of liquids: theory, estimation, experiment, and data, pp 135–405

[18] Cocchi Marina, Benedetti Pier Giuseppe De, Seeber Renato, Tassi Lorenzo, Ulrici Alessandro (1999). Development of quantitative structure- property relationships using calculated descriptors for the prediction of the physicochemical properties (n d, $$\rho$$, bp, $$\varepsilon$$, $$\eta$$) of a series of organic solvents. J Chem Inform Comput Sci.

[19] Kauffman Gregory W, Jurs Peter C (2001). Prediction of surface tension, viscosity, and thermal conductivity for common organic solvents using quantitative structure- property relationships. J Chem Inform Comput Sci.

[20] Kim Sunghwan, Thiessen Paul A, Cheng Tiejun, Zhang Jian, Gindulyte Asta, Bolton Evan E (2019). Pug-view: programmatic access to chemical annotations integrated in PubChem. J Cheminform.

[21] Dean JA et al (1999) Lange’s handbook of chemistry, 5th edn. Universitas Of Tennese Knoxville, Mc. Graw Hill Inc, New York

[22] Wasburn WE (2003). International critical tables of numerical data, physics, chemistry and technology.

[23] Rumble John R (2022). CRC handbook of chemistry and physics.

[24] Manivannan RG, Mohammad S, McCarley K, Cai T, Aichele C (2019). A new test system for distillation efficiency experiments at elevated liquid viscosities: vapor-liquid equilibrium and liquid viscosity data for cyclopentanol+ cyclohexanol. J Chem Eng Data.

[25] Chen X, Jin S, Dai Y, Jianzhou W, Guo Y, Lei Q, Fang W (2019). Densities and viscosities for the ternary system of decalin+ methylcyclohexane+ cyclopentanol and corresponding binaries at t= 293.15 to 343.15 k. J Chem Eng Data.

[26] Burk V, Pollak S, Quinones-Cisneros SE, Schmidt KAG (2021). Complementary experimental data and extended density and viscosity reference models for squalane. J Chem Eng Data.

[27] Bright Norman FH, Hutchison H, Smith D (1946). The viscosity and density of sulphuric acid and oleum. J Soc Chem Ind.

[28] Segur JB, Oberstar HE (1951). Viscosity of glycerol and its aqueous solutions. Ind Eng Chem.

[29] Landrum G et al. (2010) Rdkit. Q2.https://www.rdkit.org/. Accessed Jan – Apr 2023

[30] Ward L, Dunn A, Faghaninia A, Zimmermann NE, Bajaj S, Wang Q, Montoya J, Chen J, Bystrom K, Dylla M (2018). Matminer: an open source toolkit for materials data mining. Comput Mater Sci.

[31] Ke G, Meng Q, Finley T, Wang T, Chen W, Ma W, Ye Q, Liu T-Y, Guyon I, Luxburg UV, Bengio S, Wallach H, Fergus R, Vishwanathan S, Garnett R (2017). Lightgbm: a highly efficient gradient boosting decision tree. Advances in neural information processing systems.

[32] Chen T, Guestrin C (2016) XGBoost: a scalable tree boosting system. In: Proceedings of the 22nd ACM SIGKDD International Conference on Knowledge Discovery and Data Mining, KDD’16, ACM, New York. pp 785–794. 10.1145/2939672.2939785

[33] Pedregosa F, Varoquaux G, Gramfort A, Michel V, Thirion B, Grisel O, Blondel M, Prettenhofer P, Weiss R, Dubourg V, Vanderplas J, Passos A, Cournapeau D, Brucher M, Perrot M, Duchesnay E (2011). Scikit-learn: machine learning in Python. J Mach Learn Res.

[34] Yang Y, Yao K, Repasky MP, Leswing K, Abel R, Shoichet BK, Jerome SV (2021). Efficient exploration of chemical space with docking and deep learning. J Chem Theor Comput.

[35] Benchmark study of deepautoqsar, chemprop, and deeppurpose on the admet subset of the therapeutic data commons (2022) https://www.schrodinger.com/sites/default/files/22_086_machine_learning_white_paper_r4-1.pdf. Accessed 4 May 2024

[36] Kipf TN, Welling M (2016) Semi-supervised classification with graph convolutional networks. arXiv preprint arXiv:1609.02907

[37] Duvenaud DK, Maclaurin D, Iparraguirre J, Bombarell R, Hirzel T, Aspuru-Guzik A, Adams RP (2015) Convolutional networks on graphs for learning molecular fingerprints. In: Advances in neural information processing systems, p 28

[38] Knyazev B, Taylor GW, Amer M (2019) Understanding attention and generalization in graph neural networks. In: Advances in neural information processing systems, p 32

[39] Hamilton W, Ying Z, Leskovec J (2017) Inductive representation learning on large graphs. In: Advances in neural information processing systems, p 30

[40] Xu K, Hu W, Leskovec J, Jegelka S (2018) How powerful are graph neural networks? arXiv preprint arXiv:1810.00826,

[41] Lee J, Lee I, Kang J (2019) Self-attention graph pooling. In: International conference on machine learning, PMLR. pp 3734–3743

[42] Diehl F (2019) Edge contraction pooling for graph neural networks. arXiv preprint arXiv:1905.10990

[43] Vinyals O, Bengio S, Kudlur M (2015) Order matters: sequence to sequence for sets. arXiv preprint arXiv:1511.06391

[44] Zhang M, Cui Z, Neumann M, Chen Y (2018) An end-to-end deep learning architecture for graph classification. In: Proceedings of the AAAI conference on artificial intelligence, vol. 32

[45] Paszke A, Gross S, Massa F, Lerer A, Bradbury J, Chanan G, Killeen T, Lin Z, Gimelshein N, Antiga L, Desmaison A, Kopf A, Yang E, DeVito Z, Raison M, Tejani A, Chilamkurthy S, Steiner B, Fang L, Bai J, Chintala S (2019) Pytorch: an imperative style, high-performance deep learning library. In Advances in Neural Information Processing Systems vol. 32. Curran Associates, Inc., pp 8024–8035. http://papers.neurips.cc/paper/9015-pytorch-an-imperative-style-high-performance-deep-learning-library.pdf. Accessed Jan – Apr 2023

[46] Version 2022–2 Materials Science Suite (2022) Schrödinger, llc, New York. https://www.schrodinger.com/platform/materials-science. Accessed Jan – Apr 2023

[47] Bowers KJ, Chow E, Xu H, Dror RO, Eastwood MP, Gregersen BA, Klepeis JL, Kolossvary I, Moraes MA, Sacerdoti FD, et al (2006) Scalable algorithms for molecular dynamics simulations on commodity clusters. In: Proceedings of the 2006 ACM/IEEE Conference on Supercomputing, p. 84

[48] Afzal MAF, Browning AR, Goldberg A, Halls MD, Gavartin JL, Morisato T, Hughes TF, Giesen DJ, Goose JE (2020). High-throughput molecular dynamics simulations and validation of thermophysical properties of polymers for various applications. ACS Appl Polym Mater.

[49] Chao L, Chuanjie W, Ghoreishi D, Chen W, Wang L, Damm W, Ross GA, Dahlgren MK, Russell E, Von Bargen CD (2021). Opls4: improving force field accuracy on challenging regimes of chemical space. J Chem Theor Comput.

[50] Zahrt AF, Henle JJ, Denmark SE (2020). Cautionary guidelines for machine learning studies with combinatorial datasets. ACS Comb Sci.

[51] Dixon SL, Duan J, Smith E, Von Bargen CD, Sherman W, Repasky MP (2016). AutoQSAR: an automated machine learning tool for best-practice quantitative structure-activity relationship modeling. Future Med Chem.

[52] Lundberg SM, Lee S-I (2017) A unified approach to interpreting model predictions. In: Guyon I, Luxburg UV, Bengio S, Wallach H, Fergus R, Vishwanathan S, Garnett R (eds) Advances in Neural Information Processing Systems vol. 30. Curran Associates, Inc., pp 4765–4774. http://papers.nips.cc/paper/7062-a-unified-approach-to-interpreting-model-predictions.pdf. Accessed Jan – Apr 2023

[53] Lundberg SM, Erion G, Chen H, DeGrave A, Prutkin JM, Nair B, Katz R, Himmelfarb J, Bansal N, Lee S-I (2020). From local explanations to global understanding with explainable ai for trees. Nat Mach Intell.

[54] Molnar C (2022) Interpretable machine learning. 2nd edn. https://christophm.github.io/interpretable-ml-book. Accessed Jan – Apr 2023

[55] Rodríguez-Pérez R, Bajorath J (2019). Interpretation of compound activity predictions from complex machine learning models using local approximations and shapley values. J Med Chem.

[56] Bannigan P, Bao Z, Hickman RJ, Aldeghi M, Häse F, Aspuru-Guzik A, Allen C (2023). Machine learning models to accelerate the design of polymeric long-acting injectables. Nat Commun.

[57] Afzal MAF, Sonpal A, Haghighatlari M, Schultz AJ, Hachmann J (2019). A deep neural network model for packing density predictions and its application in the study of 1.5 million organic molecules. Chem Sci.

[58] Wellawatte GP, Gandhi HA, Seshadri A, White AD (2022). A perspective on explanations of molecular prediction models. J Chem Theor Comput.

[59] Sanchez-Lengeling B, Wei J, Lee B, Reif E, Wang P, Qian W, McCloskey K, Colwell L, Wiltschko A (2020). Evaluating attribution for graph neural networks. Adv Neural Inf Process Syst.

[60] Huang Q, Yamada M, Tian Y, Singh D, Chang Y (2022) Graphlime: local interpretable model explanations for graph neural networks. IEEE Trans Knowl Data Eng

[61] Weber JK, Morrone JA, Bagchi S, Estrada JD, Pabon SK, Zhang L, Cornell WD (2022). Simplified, interpretable graph convolutional neural networks for small molecule activity prediction. J Comput-Aided Mol Des.

[62] Rodríguez-Pérez R, Bajorath J (2020). Interpretation of machine learning models using shapley values: application to compound potency and multi-target activity predictions. J Comput-Aided Mol Des.

[63] Bonchev D, Trinajstić N (1977). Information theory, distance matrix, and molecular branching. J Chem Phys.

[64] Qun-Fang L, Yu-Chun H, Rui-Sen L (1997). Correlation of viscosities of pure liquids in a wide temperature range. Fluid Ph Equilib.

[65] Miller AA (1963). “Free volume” and the viscosity of liquid water. J Chem Phys.

[66] Kim SC, Oyakhire ST, Athanitis C, Wang J, Zhang Z, Zhang W, Boyle DT, Kim MS, Yu Z, Gao X (2023). Data-driven electrolyte design for lithium metal anodes. Proc Natl Acad Sci.

