# Supplementary material for: Advancing material property prediction: using physics-informed machine learning models for viscosity
Source: J Cheminform. 2024 Mar 14;16:31. doi: 10.1186/s13321-024-00820-5 (PMC10938832; doi:10.1186/s13321-024-00820-5)
Supplement: Supplementary file 1 — Additional file 1. This file contains details of the curated viscosity dataset, how molecular dynamics descriptors are computed, the correlation between top descriptors and viscosity, the stability of molecular dynamics descriptors, and hyperparameters for QSPR models. [file 13321_2024_820_MOESM1_ESM.pdf]

Additional file

Advancing Material Property Prediction: Using Physics-Informed Machine Learning Models for Viscosity

Alex K. Chew[a], Matthew Sender[b], Zachary Kaplan[a], Anand Chandrasekaran[a], Jackson Chief Elk[b], Andrea R. Browning[b], H. Shaun Kwak[b], Mathew D. Halls[c], Mohammad Atif Faiz Afzal[b,*]

[a] *Schrödinger, Inc., New York 10036, United States*
[b] *Schrödinger, Inc., Portland, Oregon 97204, United States*
[c] *Schrödinger, Inc., San Diego, California 92121, United States*

Contents

S1 Viscosity dataset **2**
S1.1 Sources . . . 2
S1.2 Additional dataset analysis . . . 2

S2 Descriptors **3**
S2.1 Molecular dynamics descriptors . . . 3
S2.2 Descriptor correlation to viscosity . . . 4
S2.3 Stability of MD descriptors . . . 5

S3 QSPR models **7**
S3.1 Data splitting . . . 7
S3.2 Hyperparameters . . . 9
S3.3 Performance of QSPR Models . . . 11
S3.4 Percent change in test RMSE when including MD descriptors . . . 13
S3.5 Impact of noise from MD descriptors on QSPR models . . . 13

S4 Data availability **15**
S4.1 Data . . . 15
S4.2 Model . . . 16
S4.3 Prediction of solvents related to battery electrolyte design . . . 17

*Corresponding author
Email address: atif.afzal@schrodinger.com (Mohammad Atif Faiz Afzal)

S1. Viscosity dataset

S1.1. Sources

Table S1 summarizes the sources of viscosity that were extracted to generate the 4,440 curated dataset.

Table S1: Literature sources used to extract temperature-dependent viscosities. This table summarizes the number of instances, unique molecules, experimental temperature (T_{exp}) range, viscosity range, and log-viscosity range for each source. Sources are listed in descending order relative to the number of instances.

Source	# instances	# unique molecules	T_{exp} range (K)	μ range (cP)	Log μ range
[1]	1403	298	(227.45, 403.41)	(0.15, 25.05)	(-0.82, 1.40)
[2]	1172	185	(233.10, 404.10)	(0.10, 26.52)	(-1.00, 1.42)
[3]	858	233	(248.15, 373.15)	(0.18, 25.40)	(-0.74, 1.40)
[4]	338	220	(273.15, 403.15)	(0.22, 26.09)	(-0.66, 1.42)
[5]	200	185	(230.32, 393.15)	(0.14, 25.60)	(-0.86, 1.41)
[6]	192	192	(298.15, 298.15)	(0.23, 19.90)	(-0.64, 1.30)
[7]	177	176	(278.15, 378.15)	(0.18, 19.90)	(-0.74, 1.30)
[8]	45	45	(298.15, 298.15)	(0.34, 13.30)	(-0.47, 1.12)
[9]	22	22	(291.15, 336.15)	(0.24, 9.79)	(-0.62, 0.99)
[10]	15	1	(303.15, 373.15)	(1.18, 8.20)	(0.07, 0.91)
[11]	7	1	(293.15, 343.15)	(2.53, 11.88)	(0.40, 1.07)
[12]	6	1	(303.15, 353.15)	(4.69, 22.50)	(0.67, 1.35)
[13]	4	1	(298.15, 353.15)	(5.61, 24.77)	(0.75, 1.39)
[14]	1	1	(363.15, 363.15)	(21.30, 21.30)	(1.33, 1.33)
Summary	4440	1005	(227.45, 404.10)	(0.10, 26.52)	(-1.00, 1.42)

S1.2. Additional dataset analysis

We further analyzed the viscosity dataset to see how well our curated dataset accounts for temperature information within the model. Figure S1 shows a histogram of the number of temperature examples per molecule for the curated viscosity dataset. Of the entire dataset, 478 unique molecules have a single temperature-viscosity example, whereas the remaining 527 unique molecules have multiple temperature-viscosity examples. The histogram shows a general decrease in molecules with multiple temperature-viscosity examples with molecules that can have up to ∼40 temperature-viscosity examples. In sum, the dataset has a balance of both chemical heterogeneity and temperature dependence of viscosity, which are both

important for developing generalizable quantitative structure-property relationships (QSPR) models for predicting viscosity.

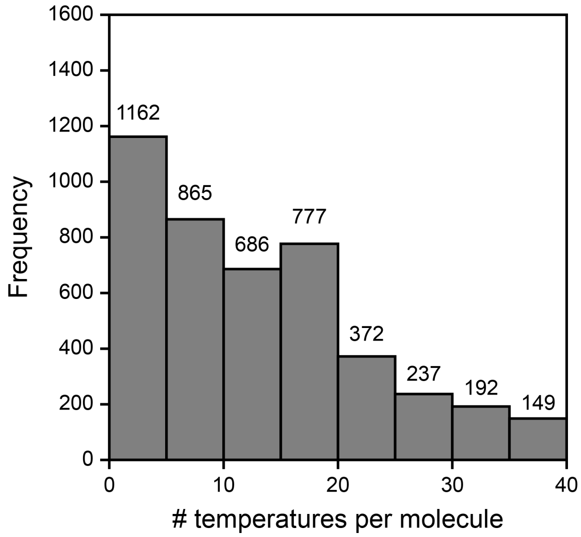

Figure S1: Histogram of number of temperature examples per molecule for the viscosity dataset.

S2. Descriptors

S2.1. Molecular dynamics descriptors

We computed eight descriptors from molecular dynamics (MD) simulations:

1. Packing density (MD_density): Calculated by taking the average cell size of the last 20% of a stage from the equilibration protocol which includes 10 ns NPT at T_{exp} and pressure of 1 atm. MD_density has units of g/cm^3.

2. Percentage free volume (MD_FV): Extracted by taking the free volume percentage from the output of the production run, which includes 20 ns NVT at T_{exp} and pressure of 1 atm. We used the spacing between grid points as 0.25 Å for the free volume calculation. MD_FV is unitless.

3. Radius of gyration of the molecule (MD_Rg): Calculated by taking the average Rg of all molecules across all the snapshots in the last 10 ns of the production run, which is 100 snapshots in total. MD_Rg has units of Å.

4. Heat of vaporization (MD_HV): Calculated from the energy of the periodic unit cell (E_{cell}) minus the sum of the N individual molecules, E_i, averaged over the last 10 ns of production NVT trajectory, as

$$\Delta H_V = \left\langle E_{cell} - \sum_i E_i \right\rangle + RT_{exp} \tag{S1}$$

R is a gas constant with a value of 1.9872036×10^{-3} kcal K^{-1} mol^{-1}, and T_{exp} is the experimental temperature. MD_HV has units of kcal/mol.

5. Hansen solubility parameters (MD_SP, MD_SP_E, and MD_SP_V): The solubility parameter (MD_SP) is calculated using the equation:

$$\delta = \left[\frac{(\Delta H_V - RT)]}{V_M} \right]^{\frac{1}{2}} \tag{S2}$$

where ΔH_V and V_M is the molar volume. We also compute MD_SP_E and MD_SP_V by taking the van der Waals (vdW) and electrostatic contributions to the above equation, respectively. The values are averaged over the last 10 ns of the production NVT trajectory. MD_SP, MD_SP_E, and MD_SP_V have units of $\mathrm{MPa}^{(1/2)}$

6. Root-mean-square displacement (MD_RMSD): We compute the root-mean-square displacement of all the molecules over the last 10 ns of production NVT trajectory. MD_RMSD has units of Å.

S2.2. Descriptor correlation to viscosity

We investigated whether the top descriptors as observed in Fig. 5 of the main text are correlated to viscosity. Fig. S2 shows a scatter plot between log viscosity versus top descriptors for the entire dataset with Pearson's r correlation coefficient reported on the lower right. Fig. S2A shows that log viscosity has low correlation with inverse temperature when structural information is omitted, which means that temperature information alone is not sufficient to capture viscosity trends across different chemical species. RD_PEOE_VSA1 was identified as a top RDKit descriptor relevant to log viscosity in the main text (Fig. 5), but this descriptor alone has a low Pearson's r of 0.42 relative to log viscosity (see Fig. S2B). MD-derived heat of vaporization (MD_HV) has a higher Pearson's r of 0.76 relative to RD_PEOE_VSA1 (see Fig. S2C), which suggests that the MD descriptors may be better at capturing trends in viscosity as compared to two-dimensional structural descriptors. The remaining top MD descriptors (MD_Rg, MD_RMSD, and MD_FV) have a negative correlation to log viscosity (Fig. S2D-F) and do not correlate as strongly to log viscosity as compared to MD_HV.

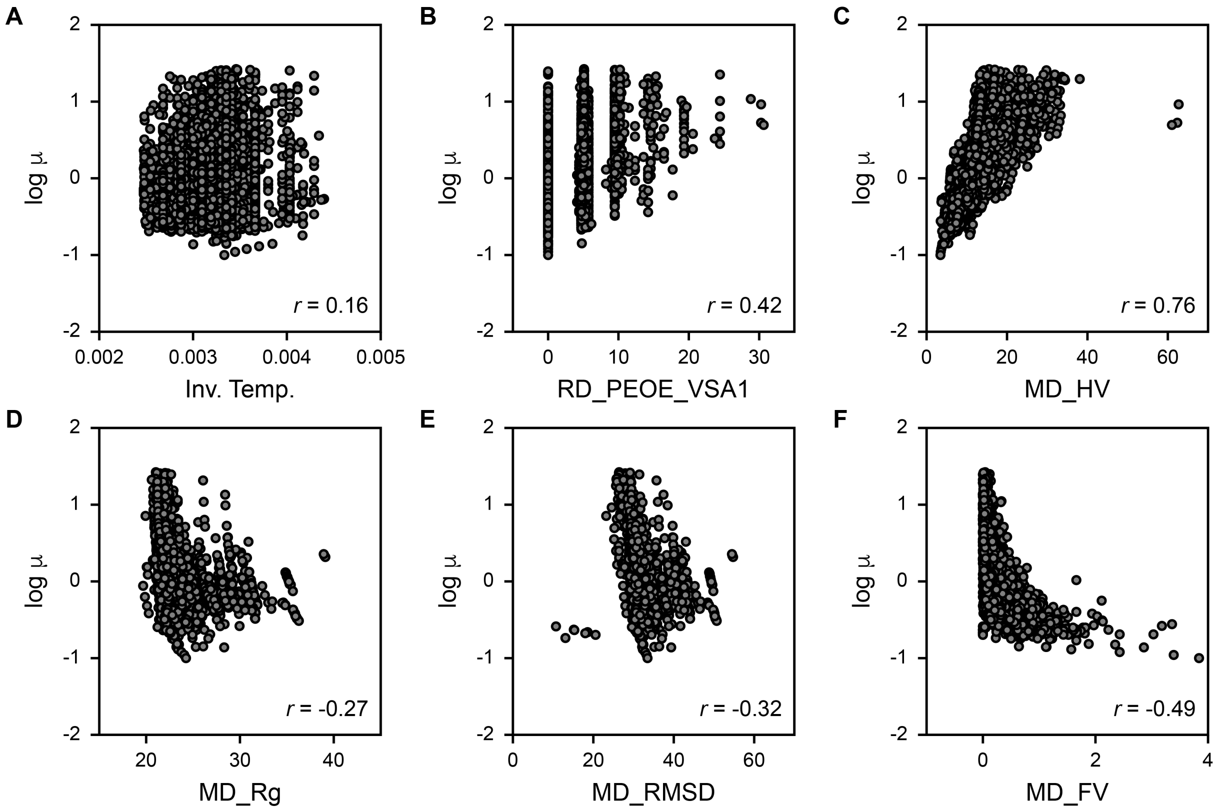

Figure S2: Correlation of top descriptors to viscosity. Scatter plot of log-scale viscosities (μ) to (**A**) experimental inverse temperature (Inv. Temp.), (**B**) "MOE-like" charge van der Waal's surface area descriptor (RD_PEOE_VSA1), (**C**) heat of vaporization (MD_HV), (**D**) radius of gyration of the molecule (MD_Rg), (**E**) root-mean-square displacement (MD_RMSD), and (**F**) percentage free volume (MD_FV). Pearson's r correlation coefficient between log μ and descriptor is shown in the lower right hand of each plot.

S2.3. Stability of MD descriptors

We evaluated the stability of MD descriptors by investigating these descriptors, simulation energies, and simulation temperatures as a function of simulation time for low and high viscosity examples. As a low viscosity example, we arbitrarily selected (Z)-1,2-Dibromoethene at a temperature of 274 K that has an experimental μ of 1.2 cP. Fig. S3A shows MD_Rg, MD_HV, and MD_RMSD versus simulation time for (Z)-1,2-Dibromoethene, which shows that MD descriptors are well-converged after 10 ns. Fig. S3B shows the simulation potential energies, kinetic energies, and temperatures as a function of simulation time for (Z)-1,2-Dibromoethene. We observe that potential energy, kinetic energy, and temperature are well-converged in the production simulations. As a high viscosity example, we arbitrarily selected 1,2-Ethanediol at a temperature of 288 K that has an experimental μ of 26 cP. Fig. S4A shows MD_Rg, MD_HV, and MD_RMSD versus simulation time for 1,2-Ethanediol. Similar to the low viscosity example, MD descriptors are well-converged after 10 ns of simulation time. Fig. S3B shows the simulation potential energies, kinetic energies, and temperatures as a function of simulation time for 1,2-Ethanediol, which shows good convergence of energies and temperatures across the production simulation. The results from Fig. S3 and S4 highlights that even though MD struggles in directly measure

viscosities for > 5 cP, the MD descriptors extracted from short 20 ns production simulations are sufficiently converged, and they can be reliably be used for developing machine learning models to predict viscosity.

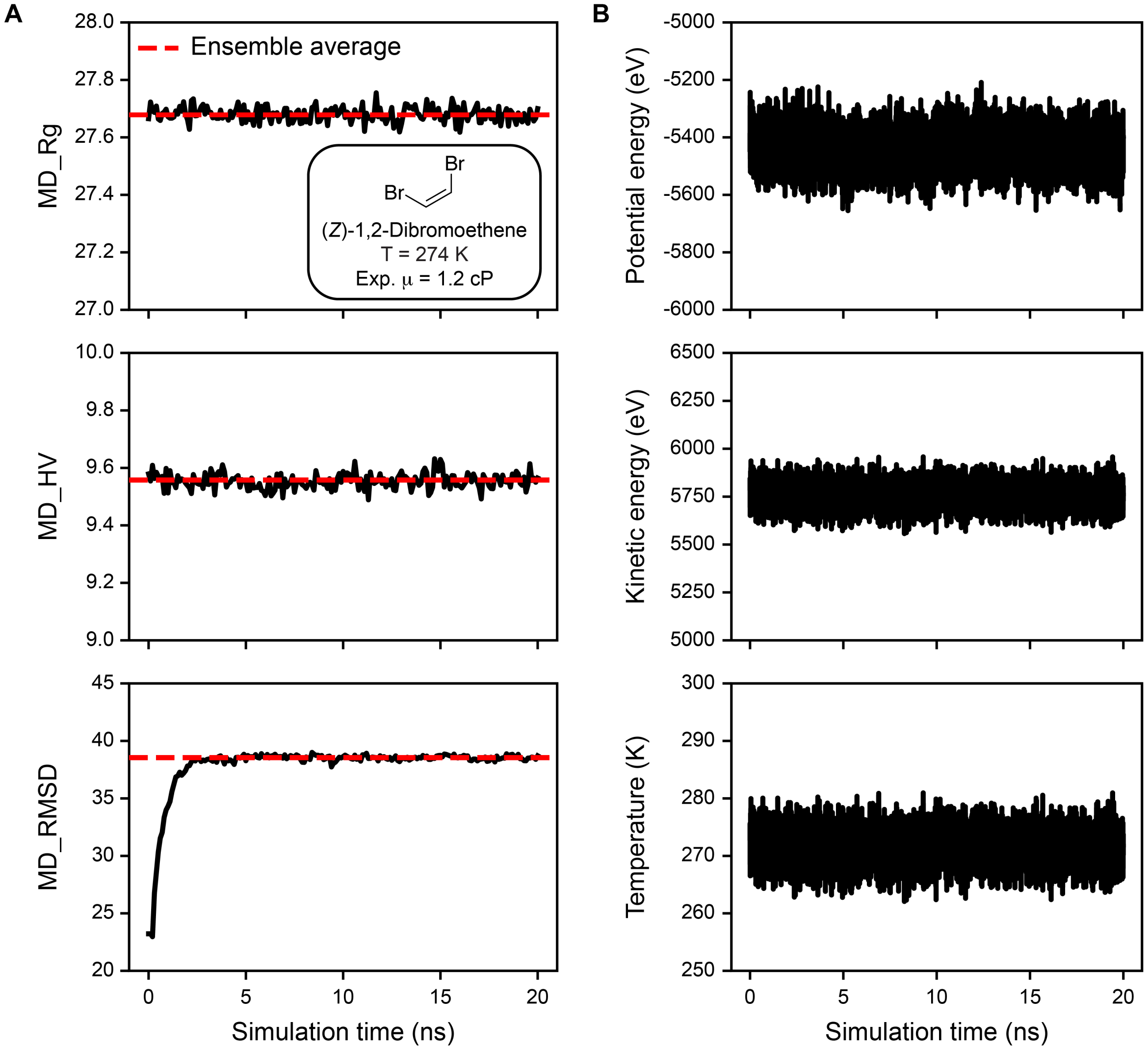

Figure S3: MD stability for a low viscosity example: (Z)-1,2-Dibromoethene at a temperature of 274 K. (**A**) Radius of gyration of the molecule (MD_Rg), heat of vaporization (MD_HV), and root-mean-square displacement (MD_RMSD) descriptors versus simulation time. (**B**) Potential energy, kinetic energy, and temperature as a function of simulation time. Ensemble average values computed using the last 10 ns of the simulation is shown as a visual guide. Molecular structure, temperature, and experimental viscosity (μ) are shown in the upper-left plot.

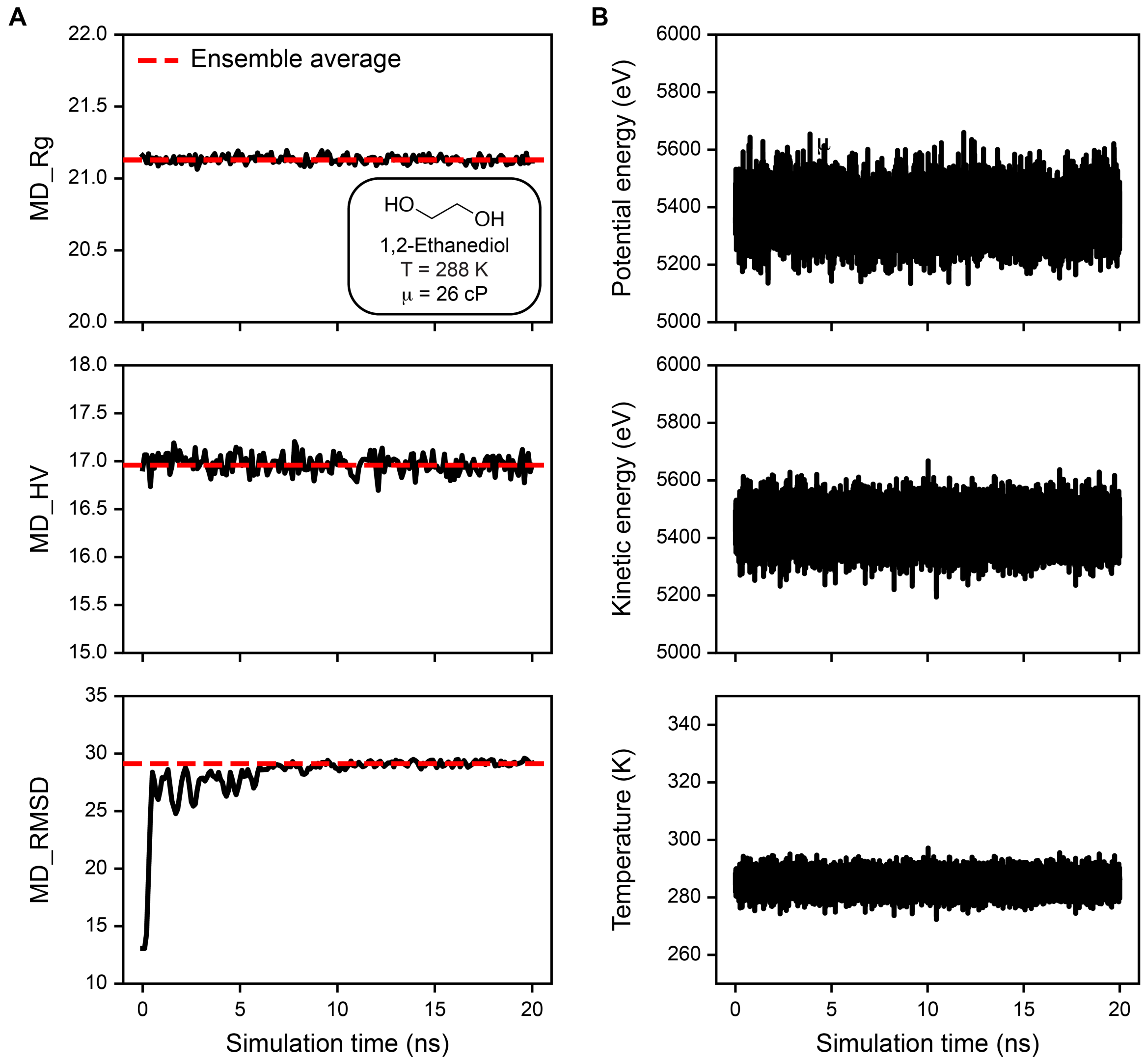

Figure S4: MD stability for a high viscosity example: 1,2-Ethanediol at a temperature of 288 K. (**A**) Radius of gyration of the molecule (MD_Rg), heat of vaporization (MD_HV), and root-mean-square displacement (MD_RMSD) descriptors versus simulation time. (**B**) Potential energy, kinetic energy, and temperature as a function of simulation time. The visual guide and format is the same as Figure S3.

S3. QSPR models

S3.1. Data splitting

Fig. S5 summarizes the data splitting workflow used to evaluate the accuracy of QSPR models in predicting viscosity. First, the dataset was split with a 80% training set and 20% testing set. A five-fold cross validation (5-CV) procedure was performed on the training set to evaluate the ability of the model to generalize on the training set. In 5-CV, the training set is partitioned into five separate sets, whereby for each of the five folds, one set is left-out as the validation set and the remaining sets are used to train the model; this procedure is repeated five times until all of the data instances are within the left-out set exactly once. Only the predictions on the left-out set are reported. After selecting the best

hyperparameters from 5-CV, the model is re-trained with the entire training set and used to predict the test set. This procedure was repeated five times for different train/test splits to reduce bias that is possible when performing only a single train/test split.

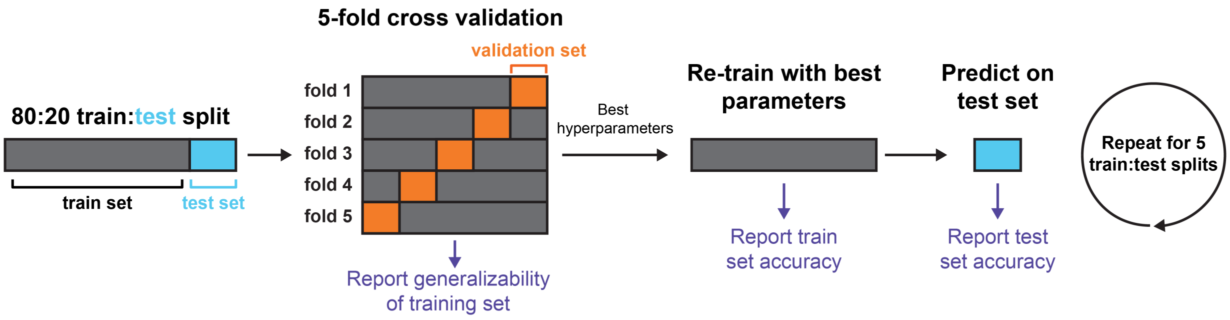

Figure S5: Data splitting workflow used to train and evaluate QSPR models in predicting viscosity.

To evaluate the impact of performing multiple train/test splits, Fig. S6 compares the 5-CV and test set root-mean-squared error (RMSE) for descriptor-based QSPR models trained to predict viscosity when performing only a single 80:20 train:test split versus an average of five 80:20 train:test splits. The 5-CV and test set RMSE is similar when performing a single train/test split as compared to averaging the performance of multiple train/test splits. The advantage of performing multiple train/test splits is that we can rigorously evaluate the QSPR model accuracy without the concern of being "lucky" for a single train/test split. Hence, we report only the performance of QSPR models evaluated from multiple train/test splits in the main text.

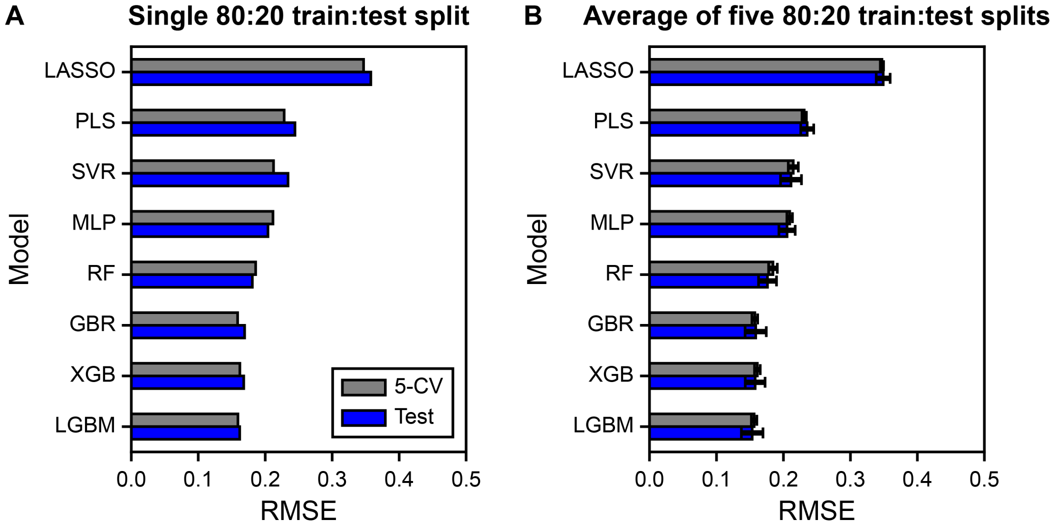

Figure S6: Comparison of 5-CV and test set RMSE for a (**A**) single versus (**B**) an average of multiple 80:20 train:test splits for descriptor-based QSPR models trained to predict viscosity. The average of multiple 80:20 train:test splits is reported in the main text as Fig. 2B. The average RMSE is reported across five out-of-sample train-test splits and the RMSE uncertainty is estimated by computing the standard deviation across the splits.

S3.2. Hyperparameters

Table S2 summarizes the hyperparameter search space for descriptor-based QSPR models. After selecting the best hyperparameters from five-fold cross validation (5-CV), the model is re-trained with the entire training set and used to predict the testing. Hyperparameters for GNN QSPR were arbitrarily fixed based on ranges from Ref. [15] and summarized in Table S3.

Table S2: Hyperparameter search space for the descriptor-based QSPR models trained to predict temperature-dependent log viscosities. Each hyperparameter name is shown with the range of values in brackets. The best parameters were identified using the scikit-learn grid search method with 2D descriptors only (2D) or combinations of 2D and MD descriptors (2D and MD). No hyperparameters were tuned for MLP models.

Model	Hyperparameter Search Space	Best Parameters (2D)	Best Parameters (2D and MD)
LGBM	n_estimators: {200.0, 500.0}<br>reg_alpha: {0.0, 0.0, 0.1, 0.1, 1.0}	n_estimators = 500.0<br>reg_alpha = 0.0	n_estimators = 500.0<br>reg_alpha = 0.0
SVR	C: {1.0, 10.0, 100.0, 1000.0, 10000.0}<br>gamma: {0.0, 0.0, 0.0, 0.0, 0.1}	C = 1000.0<br>gamma = 0.0001	C = 1000.0<br>gamma = 0.0001
GBR	max_depth: {3.0, 5.0}<br>n_estimators: {200.0, 500.0}	max_depth = 5<br>n_estimators = 500	max_depth = 5<br>n_estimators = 500
XGB	alpha: {0.0, 0.0, 0.1, 0.1, 1.0}<br>n_estimators: {200.0, 500.0}	alpha = 0.0<br>n_estimators = 500.0	alpha = 0.0<br>n_estimators = 500.0
RF	min_samples_leaf: {1.0, 10.0}<br>n_estimators: {200.0, 500.0}	min_samples_leaf = 1<br>n_estimators = 500	min_samples_leaf = 1<br>n_estimators = 200
PLS	n_components: {2.0, 4.0, 6.0, 10.0}	n_components = 10	n_components = 10
LASSO	alpha: {0.1, 0.3, 0.5, 0.7, 0.9, 1.1, 1.3, 1.5, 1.7, 1.9, 2.1}	alpha = 0.1	alpha = 0.1
MLP	-	hidden_layer_sizes = (50, 50, 50, 50)<br>max_iter = 10000<br>n_iter_no_change=1000<br>early_stopping = False<br>alpha = 0.1<br>batch_size = 500	(Same as 2D)

Table S3: Hyperparameters for graph-based QSPR models. All graph-based QSPR models had three graph convolutional hidden layers with node sizes of (128, 64, 32), followed by a fully connected layer of 128 nodes. A learning rate of 0.01, dropout of 0.25 and 500 epochs were used to train the models. This table summarizes hyperparameters specific to each individual models, as described previously in Ref. [15].

Model	Hyperparameters
GCN	Jumping Knowledge=True
TorchGraphConv	-
GraphSAGE	Jumping Knowledge=True
GIN	Jumping Knowledge=True<br>gin_train_eps = True
TopK	Jumping Knowledge=True<br>TopK ratio = 0.4
SAGPool	Jumping Knowledge=True<br>SAGPool ratio = 0.4
EdgePool	Jumping Knowledge=True
GlobalAttention	Jumping Knowledge=True
Set2Set	Jumping Knowledge=True
SortPool	Jumping Knowledge=True<br>Nodes held=6

For multilayer perceptron (MLP) models, we evaluated whether changing the hyperparameters would improve prediction accuracy by limiting the extent of overfitting that is often observed in highly parameterized neural network models. Fig. S7 compares the performance of a MLP model on a test set for an 80:20 train:test split when the `early_stopping` parameter was either True or False or when the number of hidden layers are lowered from four to two for predicting log viscosities. The `early_stopping` parameter prevents the MLP from overfitting by stopping the training after the validation coefficient of determination no longer improves, where the validation set is arbitrarily selected from 10% of the training data. Given the similar test set RMSE values when varying the `early_stopping` parameter, the results suggest that attempting to reduce overfitting by turning `early_stopping` parameter from False to True for the MLP model does not improve model performance on predicting log viscosities. We then tested whether using a shallower neural network with only two hidden layers rather than four hidden layers would improve model performance. As shown in Fig. S7, using a shallower, two-layer neural network yields similar performance to the deeper, four-layer neural network performed in the main text. Hence, the findings suggest that a shallower neural network perform similarly to a deeper neural network, but

we do not observe significant improvements in the test set RMSE. We also tested a one-layer neural network with only five neurons, which yielded an RMSE of 0.34. This value is significantly higher than those obtained from the two-layer or four-layer neural networks. Therefore, neural networks with two or more layers outperform simple single-layer neural networks for this specific viscosity dataset

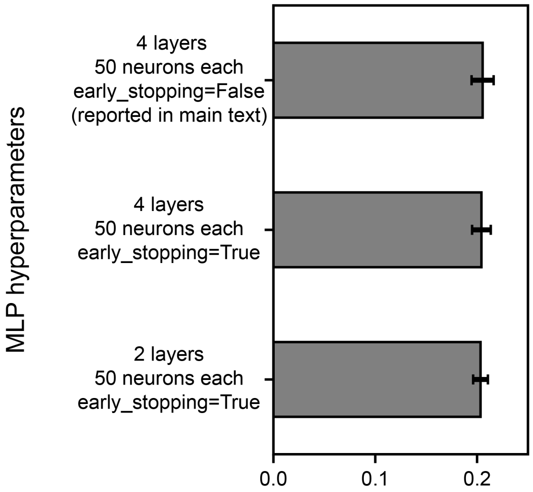

Figure S7: Test RMSE between predicted and actual log viscosities for an 80:20 train:test split using a MLP model trained with either the `early_stopping` parameter as True or False or when using a shallower neural network of two layers instead of four layers. Only 2D descriptors and external temperature were used as inputs to the model. The average test RMSE is reported, and the RMSE uncertainty is estimated by computing the standard deviation across five random, out-of-sample train-test splits.

S3.3. Performance of QSPR Models

Table S4 shows the model score, test set RMSE, and test set mean absolute error(MAE) for descriptor-based and GNN QSPR models with and without the inclusion of MD descriptors. Overall, tree-based models have the lowest test set RMSEs, such as LGBM, XGB, and GBR, followed by GNN QSPR models, such as EdgePool, GIN, SAGPool. The inclusion of MD descriptors generally improves the QSPR models in predicting viscosity with lower test set MAEs and RMSEs (or no changes).

Table S4: Model score ($Score_M$), test set RMSE, and test set MAE with and without MD descriptors for descriptor and GNN QSPR models trained to predict the viscosity dataset with 80:20 train:test split. Reported $Score_M$, RMSEs, and MAEs shows the average performance on predicting log viscosities for five random, out-of-sample train-test splits, and the uncertainties of these metrics are measured by the standard deviations of the train-test splits. This table is sorted by descending $Score_M$ (Without MD) values.

Model	QSPR Type	$Score_M$ (Without MD)	$Score_M$ (With MD)	Test RMSE (Without MD)	Test RMSE (With MD)	Test MAE (Without MD)	Test MAE (With MD)
LGBM	Descriptor QSPR	0.87 ± 0.03	0.88 ± 0.02	0.15 ± 0.02	0.15 ± 0.02	0.09 ± 0.01	0.09 ± 0.01
XGB	Descriptor QSPR	0.86 ± 0.02	0.88 ± 0.02	0.16 ± 0.01	0.15 ± 0.02	0.10 ± 0.01	0.09 ± 0.01
GBR	Descriptor QSPR	0.86 ± 0.02	0.88 ± 0.02	0.16 ± 0.02	0.15 ± 0.02	0.10 ± 0.01	0.09 ± 0.01
EdgePool	GNN QSPR	0.83 ± 0.01	0.86 ± 0.03	0.18 ± 0.02	0.16 ± 0.02	0.11 ± 0.01	0.10 ± 0.01
RF	Descriptor QSPR	0.83 ± 0.02	0.86 ± 0.02	0.18 ± 0.01	0.16 ± 0.02	0.11 ± 0.01	0.10 ± 0.01
GIN	GNN QSPR	0.83 ± 0.01	0.84 ± 0.06	0.17 ± 0.01	0.15 ± 0.03	0.11 ± 0.01	0.10 ± 0.01
SAGPool	GNN QSPR	0.82 ± 0.02	0.86 ± 0.01	0.18 ± 0.02	0.16 ± 0.02	0.11 ± 0.01	0.10 ± 0.01
TopK	GNN QSPR	0.81 ± 0.06	0.86 ± 0.05	0.17 ± 0.02	0.16 ± 0.02	0.11 ± 0.01	0.10 ± 0.01
GCN	GNN QSPR	0.79 ± 0.04	0.85 ± 0.02	0.18 ± 0.03	0.17 ± 0.02	0.11 ± 0.02	0.10 ± 0.01
GraphSAGE	GNN QSPR	0.79 ± 0.03	0.84 ± 0.01	0.18 ± 0.02	0.17 ± 0.01	0.11 ± 0.01	0.11 ± 0.01
MLP	Descriptor QSPR	0.78 ± 0.03	0.82 ± 0.01	0.21 ± 0.01	0.18 ± 0.01	0.13 ± 0.01	0.11 ± 0.01
SVR	Descriptor QSPR	0.77 ± 0.02	0.78 ± 0.04	0.21 ± 0.02	0.21 ± 0.02	0.15 ± 0.01	0.14 ± 0.01
Set2Set	GNN QSPR	0.77 ± 0.04	0.88 ± 0.02	0.20 ± 0.03	0.15 ± 0.01	0.12 ± 0.02	0.09 ± 0.01
PLS	Descriptor QSPR	0.74 ± 0.02	0.77 ± 0.02	0.24 ± 0.01	0.22 ± 0.01	0.16 ± 0.01	0.14 ± 0.01
GlobalAttention	GNN QSPR	0.70 ± 0.11	0.87 ± 0.02	0.22 ± 0.04	0.15 ± 0.02	0.14 ± 0.02	0.09 ± 0.01
DynamicGC	GNN QSPR	0.63 ± 0.17	0.86 ± 0.03	0.25 ± 0.06	0.16 ± 0.02	0.16 ± 0.03	0.10 ± 0.01
TorchGraphConv	GNN QSPR	0.52 ± 0.09	0.72 ± 0.02	0.31 ± 0.04	0.24 ± 0.01	0.23 ± 0.03	0.17 ± 0.01
LASSO	Descriptor QSPR	0.43 ± 0.00	0.57 ± 0.05	0.35 ± 0.01	0.30 ± 0.02	0.28 ± 0.00	0.23 ± 0.01
SortPool	GNN QSPR	0.41 ± 0.38	0.84 ± 0.05	0.32 ± 0.12	0.16 ± 0.03	0.25 ± 0.11	0.11 ± 0.01

S3.4. Percent change in test RMSE when including MD descriptors

From Fig. 4C of the main text, we observe that including MD descriptors lower test set RMSEs for LGBM and EdgePool models when predicting log viscosities. We further quantified the extent of improvement in test set RMSE with and without the inclusion of MD descriptors by computing the percent change shown in Equation S3.

$$\texttt{Percent change} = \frac{\texttt{Test RMSE with MD} - \texttt{Test RMSE without MD}}{\texttt{Test RMSE without MD}} \times 100 \qquad \text{(S3)}$$

Fig. S8 shows the percent change as a function of training size in test RMSE for LGBM and EdgePool models when including MD descriptors. Both LGBM and EdgePool models achieve at least 15% reduction in test RMSE at small data sizes with 500 training examples. As the training size increases, the percent reduction in test RMSE due to MD descriptors diminishes in magnitude and plateaus to about 10% reduction in test RMSE at 3,500 training examples. The results highlight the improvement in test RMSE across all training sizes when including just eight MD descriptors for QSPR models.

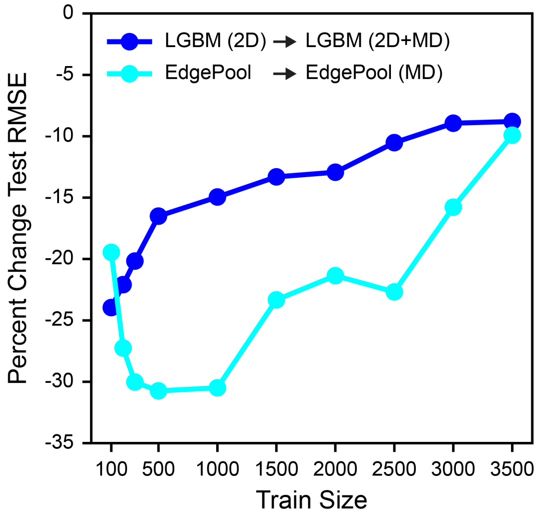

Figure S8: Percent change in test set RMSE as a function of training size for LGBM and EdgePool models when including MD descriptors.

S3.5. Impact of noise from MD descriptors on QSPR models

We investigated the impact of noisy MD descriptors by analyzing descriptors of cyclohexane as a function of temperature. Fig. S9 shows heat of vaporization computed from MD as a function of temperature for cyclohexane between the values reported in the main text (*i.e.* 1 replicate of MD simulations) as compared to the average value of 5 replicates of MD simulations. The results show that running more replicates of MD simulations can yield a better monotonic trend between MD_HV and temperature, which suggests that increasing the number of replicates for MD simulations can reduce the noise when computing MD descriptors. Given that the majority of the reported values are within the error of the

values from the 5 replicates, it suggests that a single run of MD can obtain reasonable MD descriptor estimates without requiring extensive computation time for multiple replicates.

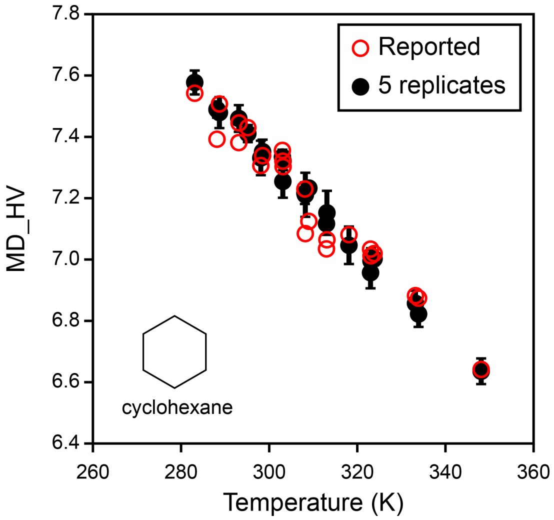

Figure S9: Simulation-derived heat of vaporization (MD_HV) versus temperature for cyclohexane using either one replicate simulation reported in the main text or five replicate simulations. For five replicate simulations, the average MD_HV value is reported and the error bar is the standard deviation of MD_HV values.

We further evaluated the impact of noise from MD descriptors on QSPR model predictions by perturbing MD_HV values for cyclohexane at T = 298.5 K while keeping all other MD descriptors fixed and observing how viscosity predictions change as a function of MD_HV. We perturbed MD_HV by adding random numbers from a normal distribution with a mean MD_HV value of 7.34 and standard deviation of 0.04. The average and standard deviation value of MD_HV was estimated from the five MD simulation replicates for cyclohexane as described in Fig. S9. Fig. S10 shows the predicted log viscosity as a function of perturbed MD_HV values for cyclohexane at T = 298.5 K for LGBM (2D+MD) and EdgePool (MD) models that were trained with the entire viscosity dataset. The results show that adding noise to MD_HV minimally impacts the prediction of log viscosities, where variations in MD_HV with a standard deviation of 0.04 results in a standard deviations of ∼0.002-0.003 in predicted log viscosities for LGBM and EdgePool models.

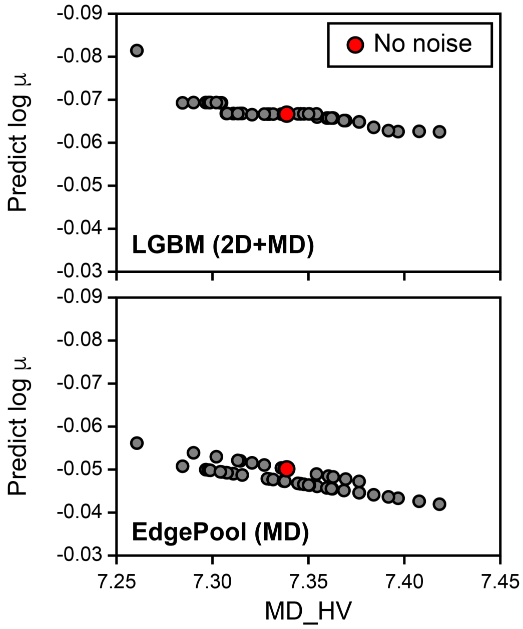

Figure S10: Predicted log viscosities (μ) when perturbing simulation-derived heat of vaporization (MD_HV) for cylohexane at T = 298.5 K for LGBM (2D+MD) and EdgePool (MD) models. Predicted log μ for the original MD_HV value with no noise is shown as a red point. Fifty perturbations were performed by adding a random numbers to MD_HV from a normal distribution with a mean MD_HV value of 7.34 and standard deviation of 0.04.

S4. Data availability

S4.1. Data

The viscosity dataset is provided in a CSV file format under the Creative Commons Non-Commercial 4.0 International (CC-BY-NC 4.0) Attribution License. This license allows for the use of the dataset and the creation of adaptations, exclusively for non-commercial purposes, provided that appropriate credit is given.

Due to copyright restrictions, we cannot release the viscosity data from Ref. [3]; hence, we provide a subset of the dataset without data from Ref. [3]. The supporting information contains a spreadsheet containing 3,582 examples of temperature-dependent viscosities with the columns described in Table S5.

Table S5: Description of columns for the curated viscosity dataset.

Column	Description
Index	Arbitrary index for each row.
Name	Name of the molecule (may vary slightly depending on the source).
CANON_SMILES	Canonical SMILES
Temperature (K)	Experimental temperature in Kelvin.
Inverse temperature (1/K)	Inverse experimental temperature in K^{-1}.
Viscosity (cP)	Experimental viscosity in centipose.
log(Viscosity)	Logarithm with base 10 of the viscosity.
MD_density	MD-computed density in g/cm^3.
MD_FV	MD-computed percentage free volume (unitless).
MD_Rg	MD-computed radius of gyration of the molecule in Å.
MD_SP	MD-computed solubility parameter using Equation S2 with units of $MPa^{(1/2)}$.
MD_SP_E	MD-computed solubility parameter (electrostatic contribution) in $MPa^{(1/2)}$.
MD_SP_V	MD-computed solubility parameter (van der Waals contribution) in $MPa^{(1/2)}$.
MD_HV	MD-computed heat of vaporization in kcal/mol.
MD_RMSD	MD-computed root-mean-square displacement in Å.
Reference	Source of the data value.

S4.2. Model

A pre-trained LGBM model is made available in the link below:

https://doi.org/10.5281/zenodo.8350587.

The available LGBM model was trained with the subset dataset described in Section S4.1 using all 2D descriptors and inverse temperature as model inputs. Fig. S11 compares the available LGBM model against one that is trained with the full dataset (see Fig. 2D of the main text). Fig. S11A compares the test set RMSE for an 80:20 train:test split for a LGBM model trained when using either the full dataset or the subset dataset. The test RMSEs for a LGBM model trained with either datasets are very similar, suggesting that the LGBM model accurately predicts the test set even when using a subset dataset. Fig. S11B shows a parity plot of predicted versus actual log viscosities for the training and testing sets for a LGBM model trained with the subset dataset. The resulting train/test R^2 and RMSE is comparable to that observed in Fig. 2D of the main text. In sum, the available LGBM model that is trained with the subset dataset demonstrates comparable accuracies as the one reported in the main text.

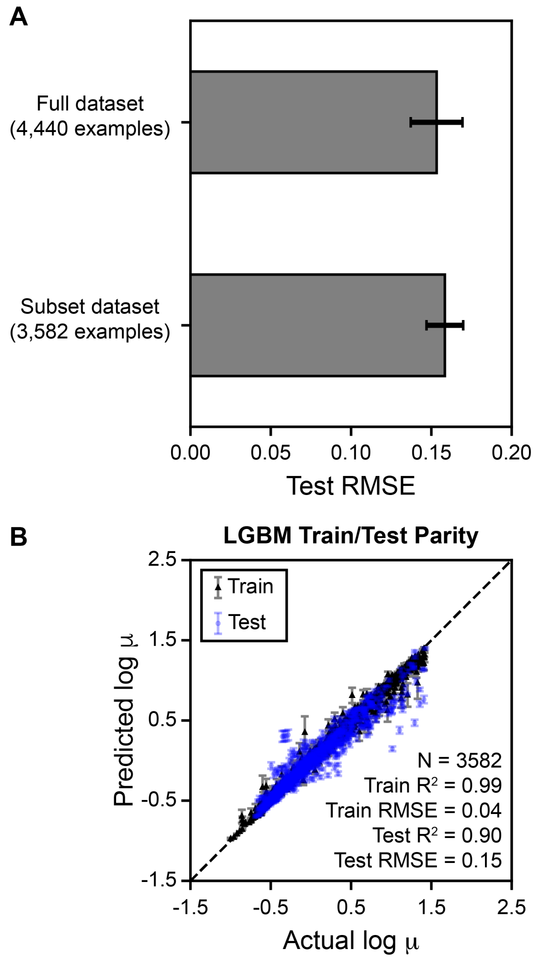

Figure S11: (**A**) Test RMSE comparison for an 80:20 train:test split using a LGBM model trained with either the full dataset described in the main text or the subset dataset described in Section S4.1. The average test RMSE is reported, and the RMSE uncertainty is estimated by computing the standard deviation across five random, out-of-sample train-test splits. (**B**) Parity plot between predicted and actual log viscosity for an 80:20 train:test split for the LGBM model trained with the subset dataset. The statistics and visual guides are the same as Fig. 2D of the main text.

S4.3. Prediction of solvents related to battery electrolyte design

Predictions of 50 solvents related to battery electrolyte design for lithium metal anodes from Ref. [16] are made available using an EdgePool model trained with the entire viscosity dataset and without the inclusion of MD descriptors. The supporting information contains a spreadsheet with 650 rows of predicted log viscosities as a function of temperature from 270 K to 330 K. Table S6 summarizes the column names for this spreadsheet.

Table S6: Description of columns for the predictions of solvents from Ref. [16] when using an EdgePool model trained with the entire viscosity dataset and without the inclusion of MD descriptors. Predictions were performed for temperature ranging from 270 K to 330 K.

Column	Description
Index	Arbitrary index for each row.
Solvent Name	Solvent name, which corresponds to the names from [16].
CANON_SMILES	Canonical SMILES
Temperature (K)	Experimental temperature in Kelvin.
Inverse temperature (1/K)	Inverse experimental temperature in K^{-1}.
EdgePool_log(Viscosity)_pred	Predicted log viscosities when using an EdgePool model without MD descriptors.
is_within_training	True if the structure is within the viscosity dataset used to train the model.

References

[1] WE Wasburn. International critical tables of numerical data, physics, chemistry and technology. 1st electronic edition. *Knovel: Norwich, NY*, 2003.

[2] Diego Alonso Saldana, Laurie Starck, Pascal Mougin, Bernard Rousseau, Nicolas Ferrando, and Benoit Creton. Prediction of density and viscosity of biofuel compounds using machine learning methods. *Energy & fuels*, 26(4):2416–2426, 2012.

[3] John R Rumble. Crc handbook of chemistry and physics, 103rd ed., 2022.

[4] A Dean John et al. Lange's handbook of chemistry. In *Universitas Of Tennese Knoxville, Fifthenth Edition, Mc. Graw Hill Inc, New York. Conference*, 1999.

[5] Sunghwan Kim, Paul A Thiessen, Tiejun Cheng, Jian Zhang, Asta Gindulyte, and Evan E Bolton. Pug-view: programmatic access to chemical annotations integrated in pubchem. *Journal of cheminformatics*, 11(1):1–11, 2019.

[6] Valentin Goussard, François Duprat, Jean-Luc Ploix, Gérard Dreyfus, Véronique Nardello-Rataj, and Jean-Marie Aubry. A new machine-learning tool for fast estimation of liquid viscosity. application to cosmetic oils. *Journal of Chemical Information and Modeling*, 60(4):2012–2023, 2020.

[7] Dabir S Viswanath, Tushar K Ghosh, Dasika HL Prasad, Nidamarty VK Dutt, Kalipatnapu Y Rani, Dabir S Viswanath, Tushar K Ghosh, Dasika HL Prasad, Nidamarty VK Dutt, and Kalipatnapu Y Rani. Correlations and estimation of pure liquid viscosity. *Viscosity of Liquids: Theory, Estimation, Experiment, and Data*, pages 135–405, 2007.

[8] Gregory W Kauffman and Peter C Jurs. Prediction of surface tension, viscosity, and thermal conductivity for common organic solvents using quantitative structure- property relationships. *Journal of chemical information and computer sciences*, 41(2):408–418, 2001.

[9] Marina Cocchi, Pier Giuseppe De Benedetti, Renato Seeber, Lorenzo Tassi, and Alessandro Ulrici. Development of quantitative structure- property relationships using calculated descriptors for the prediction of the physicochemical properties (n d, ρ, bp, ε, η) of a series of organic solvents. *Journal of chemical information and computer sciences*, 39(6):1190–1203, 1999.

[10] Raj Ganesh Manivannan, Sayeed Mohammad, Ken McCarley, Tony Cai, and Clint Aichele. A new test system for distillation efficiency experiments at elevated liquid viscosities: vapor–liquid equilibrium and liquid viscosity data for cyclopentanol+ cyclohexanol. *Journal of Chemical & Engineering Data*, 64(2):696–705, 2019.

[11] Xiaoyi Chen, Shenda Jin, Yitong Dai, Jianzhou Wu, Yongsheng Guo, Qunfang Lei, and Wenjun Fang. Densities and viscosities for the ternary system of decalin+ methylcyclohexane+ cyclopentanol and corresponding binaries at t= 293.15 to 343.15 k. *Journal of Chemical & Engineering Data*, 64(4):1414–1424, 2019.

[12] Vincent Burk, Stefan Pollak, Sergio E Quinones-Cisneros, and Kurt AG Schmidt. Complementary experimental data and extended density and viscosity reference models for squalane. *Journal of Chemical & Engineering Data*, 66(5):1992–2005, 2021.

[13] Norman FH Bright, H Hutchison, and D Smith. The viscosity and density of sulphuric acid and oleum. *Journal of the Society of Chemical Industry*, 65(12):385–388, 1946.

[14] John Bartlett Segur and Helen E Oberstar. Viscosity of glycerol and its aqueous solutions. *Industrial & Engineering Chemistry*, 43(9):2117–2120, 1951.

[15] Benchmark study of deepautoqsar, chemprop, and deeppurpose on the admet subset of the therapeutic data commons. https://www.schrodinger.com/sites/default/files/22_086_machine_learning_white_paper_r4-1.pdf, 2022. Accessed: 2024-05-04.

[16] Sang Cheol Kim, Solomon T Oyakhire, Constantine Athanitis, Jingyang Wang, Zewen Zhang, Wenbo Zhang, David T Boyle, Mun Sek Kim, Zhiao Yu, Xin Gao, et al. Data-driven electrolyte design for lithium metal anodes. *Proceedings of the National Academy of Sciences*, 120(10):e2214357120, 2023.
